# Isolation and *In Vitro* Biological Evaluation of Triterpenes from *Salacia grandifolia* Leaves

**DOI:** 10.1021/acsomega.4c04360

**Published:** 2024-07-11

**Authors:** Leila Renan Oliveira, Mateus Sá Magalhães Serafim, Diego Lanza Dias, Túlio Resende Freitas, Jonatas Santos Abrahao, Guilherme de Medeiros Antar, Bruno E. F. Mota, Adriano de Paula Sabino, Lucienir Pains Duarte, Diogo Montes Vidal, Grasiely Faria de Sousa

**Affiliations:** † Departamento de Química, Universidade Federal de Minas Gerais, Belo Horizonte ,MG 31270-901, Brazil; ‡ Departamento de Microbiologia, Instituto de Ciências Biológicas, Universidade Federal de Minas Gerais, Belo Horizonte ,MG 31270-901, Brazil; § Departamento de Análises Clínicas e Toxicológicas, Faculdade de Farmácia, Universidade Federal de Minas Gerais, Belo Horizonte ,MG 31270-901, Brazil; ∥ Departamento de Ciências Agrárias e Biológicas, Universidade Federal do Espírito Santo - Campus São Mateus, São Mateus ,ES 29932-540, Brazil

## Abstract

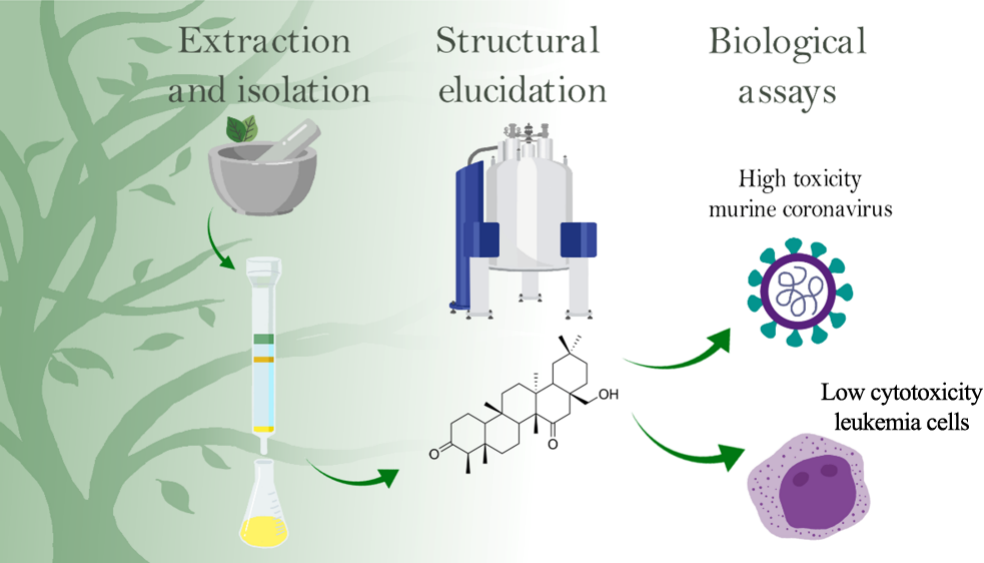

*Salacia grandifolia* is naturally found in the Atlantic Forest regions of Brazil. Despite the pharmacological potential of plants from the *Salacia* genus, phytochemical studies on this species have not been reported in literature. A new triterpene, 28-hydroxyfriedelane-3,15-dione (**1**), and seven known compounds (friedelan-3-one (**2**), friedelan-3β-ol (**3**), friedelane-3,15-dione (**4**), 15α-hydroxyfriedelan-3-one (**5**), 28-hydroxyfriedelan-3-one (**6**), 30-hydroxyfriedelan-3-one (**7**), and 29-hydroxyfriedelan-3-one (**8**)) were obtained from the hexane extract of *Salacia grandifolia* leaves. These isolated compounds and three extracts, hexane (**EH**), chloroform (**EC**), and ethyl acetate (**EAE**), were assessed for their potential biological activities, which consisted in the evaluation of antiviral activity against a murine coronavirus, mouse hepatitis virus 3 (MHV-3), antibacterial activity against the susceptible and methicillin-resistant *Staphylococcus aureus* (MRSA), and antileukemia activity against the THP-1 and K-562 cell lines. The extracts **EH** and **EAE** along with the triterpenes **1** and **6** exhibited moderate to high antiviral activity, with emphasis on **6**, which presented an EC_50_ value of 2.9 ± 0.3 μM. None of the compounds presented antibacterial activity against the tested strains. The evaluated compounds **1**, **4**, **6** and **7** exhibited low cytotoxic activity against the tested leukemia cell lines. Taken together, this study comprises an overview for the potential of the *Salacia grandifolia* biological activities, including a new isolated triterpene.

## Introduction

The Celastraceae family comprises approximately 100 genera and 1400 species, predominantly distributed in tropical and subtropical regions of the planet.^1^ The genus *Salacia* consists of approximately 200 species that have been reported to have several biological activities. For instance, *S. chinensis*, *S. oblonga*, *S. reticulata* and *S. prinoides* have shown antidiabetic activity,^2^ while *S. senegalensis* has exhibited antifungal and anti-inflammatory properties.^3^*Salacia grandifolia* (Mart.) G.Don, popularly known as “siputá,″ is naturally found in the Atlantic Forest regions of Brazil, including the States of São Paulo, Rio de Janeiro, Espírito Santo, and Bahia.^4^ Despite its pharmacological potential, there are no current phytochemical studies involving this species in the literature. Pentacyclic triterpenes are natural products derived from squalene that, due to their wide structural diversity, also exhibit several biological activities.^5^ These activities include antitumoral effects (e.g., tingenone and pristimerin), anti-inflammatory properties (e.g., celastrol), antimicrobial activity (e.g., 1α,29-dihydroxyfriedelan-3-one), cardioprotective effects (e.g., lupeol, 2α-hydroxyursolic acid), antidiabetic activity (e.g., α-amyrin), and antiviral effects (e.g., betulinic acid, oleanolic acid, and ursolic acid).^2,3,5−9^

**Figure 1:**
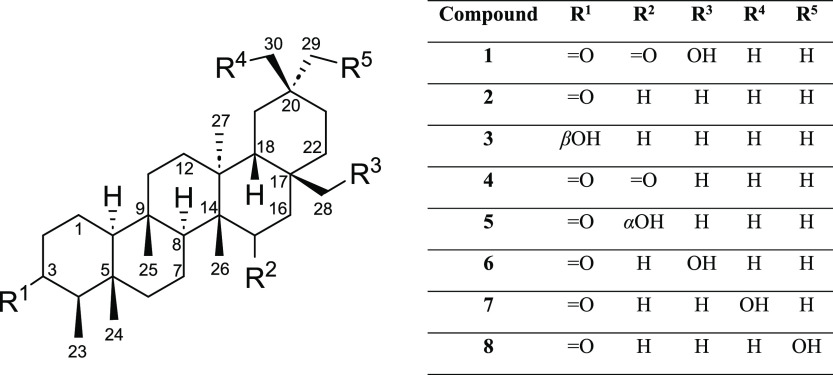
Compounds **1**–**8** isolated from *Salacia grandifolia* leaves.

In the present work, the phytochemical study of the hexane extract of *S. grandifolia* leaves led to the isolation of the new triterpene 28-hydroxyfriedelane-3,15-dione (**1**), along with seven known triterpenes: friedelan-3-one (**2**), friedelan-3β-ol (**3**), friedelane-3,15-dione (**4**), 15α-hydroxyfriedelan-3-one (**5**), 28-hydroxyfriedelan-3-one (**6**), 30-hydroxyfriedelan-3-one (**7**), and 29-hydroxyfriedelan-3-one (**8**) (Figure 1). The structural elucidation of the new triterpene **1** was established by the analysis of infrared (IR), ^1^H and ^13^C nuclear magnetic resonance (NMR), as well as two-dimensional (HSQC, HMBC, COSY, and NOESY) NMR spectra, and high-resolution electrospray ionization mass spectrometry (HR-ESI-MS). The antiviral activity of compounds **1**–**8**, as well as the hexane (**EH**), chloroform (**EC**), and ethyl acetate (**EAE**) extracts, was evaluated against the mouse hepatitis virus 3 (MHV-3), a murine coronavirus that mimics the debilitating conditions of SARS-CoV-2 infections *in vivo.*(10) Also, these compounds and extracts were assessed for their antibacterial activity against the methicillin resistant *Staphylococcus aureus* (MRSA) and susceptible *S. aureus* strains. Last, the cytotoxicity of compounds **1**, **4**, **6**, and **7** was evaluated against acute monocytic leukemia cells (THP-1) and chronic myeloid leukemia cells (K-562).

## Experimental Section

Column chromatography (CC) was carried out using silica gel 60 (70–230 mesh or 230–400 mesh, Merck, Darmstadt, Germany) as the stationary phase. The eluents used were hexane, chloroform, ethyl acetate, and methanol, either pure or in increasing polarity mixtures. Thin layer chromatography (TLC) was carried out on plates coated with silica gel 60 using a solution of 1:1 v/v vanillin (vanillin, absolute ethanol, 1% m/v) and percloric acid (3% v/v), followed by heating to 100 °C. The melting points of the isolated compounds were determined using a Microquímica apparatus, model MQAPF-302 (Microquímica, Palhoça, Brazil), without correction for normal temperature and pressure conditions. Infrared spectra were obtained using a Shimadzu IR-408 spectrometer (Shimadzu, Kyoto, Japan) with KBr pellet samples. 1D/2D NMR spectra were acquired using Bruker Avance DRX-400 and DRX-600 spectrometers (Bruker, Billerica, USA), with CDCl_3_ as solvent. Chemical shifts (δ) were measured in ppm, using tetramethylsilane (TMS; δ_H_ = δ_C_ = 0) as internal reference standard, and coupling constants (*J*) were calculated in Hz. The mass spectrum was acquired in a Shimadzu IT-TOF high-resolution mass spectrometer (Shimadzu, Kyoto, Japan) using ESI as an ionization font.

### Plant Material

The leaves of *Salacia grandifolia* were collected in Embu-Guaçu, São Paulo, Brazil, in December 2020. A voucher was collected and deposited at the Herbarium of the University of São Paulo under the number “Antar 3242”. The plant material was registered at Conselho de Gestão do Patrimônio Genético (CGEN/SisGen), Brazil, under the number AEC7E65.

### Extraction and Isolation

The leaves of *S. grandifolia* were dried at room temperature and subsequently grounded. The powder (384.5 g) was subjected to extraction by maceration with hexane, chloroform, ethyl acetate, and methanol, separately. During the partial removal of the hexane in a rotary evaporator, a solid (hexane extract solid, HES, 1.1 g, yield: 0.3%) was formed, which was filtered under reduced pressure. After filtration, all the hexane was removed, resulting in the hexane extract (HE, 7.4 g, yield: 1.9%). The removal of chloroform, ethyl acetate, and methanol yielded the chloroform (EC, 6.7 g, yield: 1.7%), ethyl acetate (EAE, 1.7 g, yield: 0.4%) and methanolic extracts (EM, 39.7 g, yield: 10.3%) extracts. HES was subjected to column chromatography (CC) using silica gel 60 (70–230 mesh), resulting in 267 fractions, which were grouped into 13 groups according to the profile on TLC (A1-A13). From these groups were obtained: friedelan-3-one (**2**) (A1, 42.1 mg, hexane/ethyl acetate 9:1 v/v); a mixture of compounds **2** and friedelan-3β-ol (**3**) (A2, 288.6 mg, hexane/ethyl acetate 9:1 v/v); 28-hydroxyfriedelan-3-one (**6**) (A6, 74.3 mg, hexane/ethyl acetate 9:1 v/v); 28-hydroxyfriedelane-3,15-dione (**1**) (A11, 47.7 mg, hexane/ethyl acetate 1:1 v/v). The remaining groups were subjected to successive CC (230–400 mesh), resulting in the following constituents: friedelane-3,15-dione (**4**) (A3, 27.4 mg, hexane/ethyl acetate 9:1 v/v); 15α-hydroxyfriedelan-3-one (**5**) (A3, 7.9 mg, hexane/ethyl acetate 9:1 v/v); 30-hydroxyfriedelan-3-one (**7**) (A8, 23.2 mg, hexane/ethyl acetate 9:1 v/v), and 29-hydroxyfriedelan-3-one (**8**) (A10, 11.1 mg, hexane/ethyl acetate 8:2 v/v). Part of the hexane extract (HE, 6.3 g) was subjected to CC using silica gel 60 (70–230 mesh), resulting in 115 fractions, which were grouped into 17 groups according to the TLC profile (B1–B17). The groups were subjected to successive CC (230–400 mesh), resulting in the following constituents: friedelan-3-one (**2**) (B4, 135.7 mg, hexane/ethyl acetate 9:1 v/v); friedelan-3β-ol (**3**) (B4, 67.1 mg, hexane/ethyl acetate 9:1 v/v and B5, 57.1 mg, hexane/ethyl acetate 9:1 v/v); friedelane-3,15-dione (**4**) (B4, 38.1 mg, hexane/ethyl acetate 9:1 v/v and B5, 35.4 mg, hexane/ethyl acetate 9:1 v/v); 30-hydroxyfriedelan-3-one (**7**) (B10, 17.7 mg, hexane/ethyl acetate 9:1 v/v).

#### 28-Hydroxyfriedelane-3,15-dione (**1**)

White solid; m.p.: decomposes at 259.6 °C; IR (KBr) ν/cm^–1^ 3530, 2934, 2868, 1708, 1700, 1458, 1388; ^1^H and ^13^C NMR: Table 1; HRMS (ESI) (negative ion mode) *m*/*z*, calcd. for C_30_H_47_O_3_^–^ [M – H]^−^ 455.3531, found: 455.3525.

**Table 1:** (600 MHz) and ^13^C (125 MHz) NMR Data of Compound **1**

**atom**	**δ**_**C**_	**δ**_**H**_	**atom**	**δ**_**C**_	**δ**_**H**_
**1**	22.2	1.68 β	**16**	47.6	1.95*β; d, J* 18.4
		1.96 α			2.99 *α;* d, *J* 18.4
**2**	41.4	2.27 α	**17**	39.3	-
		2.39 β	**18**	40.1	1.67 β
**3**	213.1		**19**	34.5	1.43
**4**	58.2	2.30 α; d, *J*7.4			1.55–1.58
**5**	42.1		**20**	27.9	
**6**	40.4	1.55	**21**	33.9 (33.85)	1.32 α
		1.71			1.39 β
**7**	21.2	1.35	**22**	31.9	1.49 β
		1.75			1.59 α
**8**	45.1	1.87 α	**23**	6.8	0.89; d, *J* 4.0
**9**	37.1		**24**	15.0	0.75; s
**10**	59.3	1.60–1.62 α	**25**	17.4	0.91; s
**11**	34.4	1.20 α	**26**	14.6	1.20; s
		1.48 β	**27**	19.4	0.99; s
**12**	29.2	1.48 β	**28**	67.6	3.69; d, *J* 10.7
		1.53 α			3.79; d, *J* 10.7
**13**	42.4		**29**	32.8	0.99; s
**14**	54.1		**30**	33.9 (33.87)	0.99; s
**15**	213.9				

### Antiviral Assay

The antiviral activity of isolated compounds and extracts was evaluated against the murine coronavirus MHV-3. Initially, the viability of L929 (mouse adipose fibroblasts; ATCC CCL-1) cells was assessed using the MTT (3-(4,5-dimethylthiazol-2-yl)-2,5-diphenyltetrazolium bromide (Thermo Fisher Scientific, USA) assay.^11^ Cells were cultured at 37 °C and 5% CO_2_ in Dulbecco’s modified Eagle medium (DMEM; Cultilab, Brazil) supplemented with 5% fetal bovine serum (FBS; Cultilab, Brazil), 100 IU/mL of penicillin (Cellofarm, Brazil), 100 μg/mL of streptomycin (Merck, Germany) and 0.25 μg/mL of amphotericin B (Cultilab, Brazil). Cells were seeded in 96-well microplates (4 × 10^4^ cells per well) and added 200 μL of DMEM with 1% de FBS containing the diluted compounds and extracts to be tested (100 to 1.56 μM or 200 to 3.125 μg/mL). Serial dilutions of DMSO were used as a vehicle control. Additionally, a 10% v/v inhibition control of DMSO was used. After 72 h of incubation under the same conditions, media was removed and 100 μL of MTT diluted in DMEM (0.5 mg/mL) were added to each well and incubated for 3 h. Last, media was removed, and 100 μL of DMSO were added to each well to solubilize the formazan crystals. The absorbance was read at 570 nm using a spectrophotometer (Versamax, Molecular Devices, EUA). The percentages of cell viability inhibition were calculated as the ratio between the absorbance of cells treated with the compounds relative to the vehicle control. Linear regression was used to calculate CC_50_ values, considering only those data for which r^2^ > 0.9. The 50% cytotoxic concentration (CC_50_) is defined as the highest concentration of a specific compound that reduces cell viability by 50%. All conditions were tested in two independent assays in triplicate.

Subsequently, MTT assays were also performed to determinate the effective concentration at which compounds were effective in protecting cells from viral infection by 50% (EC_50_), as described.^11^ Here, the same conditions as that used for CC_50_ were used, with the only difference being the concentrations of the samples (up to 8-fold dilution from the CC_50_ values for the compounds) diluted in 100 μL and added to 100 μL of viral suspension at a multiplicity of infection (MOI) of 0.1, that is, one viral particle per 10 cells in culture. Ribavirin was used as the positive control. The EC_50_ value was calculated as the percentage ratio between the absorbance of infected cells treated with the compounds and cells treated only with the vehicle. Last, the selectivity index (SI) was calculated using the ratio between CC_50_ and EC_50_. All conditions were tested in two independent assays in triplicate.

### Antibacterial Activity Evaluation

Compounds were evaluated against *S. aureus* (ATCC 29213) MRSA (ATCC 43300) strains. The antibacterial activity was evaluated with the broth microdilution method in 96-well microplates as described,^12^ in accordance to the Clinical and Laboratory Standards Institute (CLSI) protocol. Briefly, compounds were diluted in Mueller Hinton broth (MHB; Oxoid, Thermo Scientific, UK) to the concentration of 200 μM or 400 μg/mL, and 100 μL of the dilutions were added to each well and the same volume of a bacterial suspension containing 1 × 10^5^ CFU/mL, that is, 100,000 colony forming units per milliliter. Penicillin G and vancomycin were used as positive controls for *S. aureus* and MRSA, respectively. After 24 h incubation at 37 °C, the microplates were inspected visually for inhibition of bacterial growth, and the absorbance at 600 nm of each well was read using a microplate reader spectrophotometer (VersaMax, Molecular Devices, CA, USA). All conditions were tested in two independent assays in triplicate.

### Cytotoxic Assay

The cytotoxicity of compounds was evaluated against the K-562 (chronic myeloid leukemia, ATCC CCL-243) and THP-1 (acute monocytic leukemia, ATCC TIB-202) cell lines. Cell viability was determined by MTT assay using 3-(4,5-dimethylthiazol-2-yl)-2,5-diphenyltetrazolium bromide (Sigma-Aldrich, Saint Louis, EUA). For the cytotoxicity assessment, cells were planted in 96-well microplates (1 × 10^4^ cells per well) containing RPMI 1640 medium (Roswell Park Memorial Institute, Cultilab, Campinas, SP, Brazil) supplemented with 10% FBS (Cultilab, Campinas, SP, Brazil), 100 U/mL penicillin, 100 μg/mL streptomycin, and 10 mM HEPES, pH 7.4. The cells were incubated in a humidified incubator at 37 °C with 5% CO_2_ until the assay was performed. The cytotoxic assays were performed with the samples and positive controls, imatinib and cytarabine, diluted in culture medium containing 1% FBS at concentrations of 100, 10, 1, and 0.1 μg/mL. After 48 h of incubation, 100 μL of MTT salt at a concentration of 0.5 mg/mL was added to each well. The plate was then incubated for 3 h. After the incubation period, the supernatant was removed, and 50 μL of DMSO was added to each well to solubilize the formazan crystals. The absorbance per well was measured at a wavelength of 550 nm using a microplate reader (Versamax, Molecular Devices, USA). The minimum concentration that inhibited 50% of cell viability (IC_50_) in the presence of the tested compounds was determined by comparing it with cells cultured without the compounds (considered 100% viable).

## Results and Discussion

**Figure 2:**
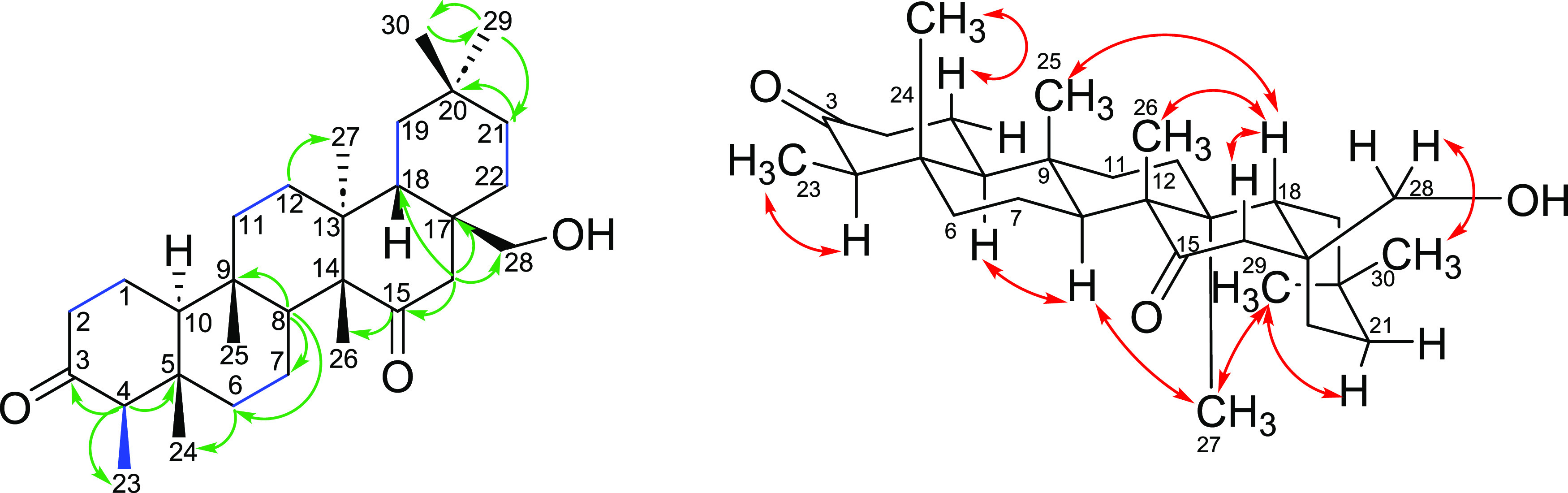
Some observed correlations in HMBC (green), COSY (blue) and NOESY (red) spectra for compound **1**.

Compound **1** was obtained as a white amorphous powder with the molecular formula C_30_H_48_O_3_. The IR spectrum displayed hydroxyl absorption at 3530 cm^–1^ and two C=O bands at 1708 and 1700 cm^–1^. ^1^H NMR spectrum showed four singlets attributed to six methyl groups (δ_H_ 0.75; 0.91; 0.99 (three overlapping signals) and 1.20), one doublet at δ_H_ 0.89 (3H, *J* = 4.0 Hz), overlapped on the signal at δ_H_ 0.91, characteristic of a friedelane skeleton 23-methyl group; two doublets at δ_H_ 3.69 (1H, *J* = 10.7 Hz) and 3.79 (1H, *J* = 10.7 Hz), characteristic of hydroxymethylene hydrogen atoms, and two doublets at δ_H_ 2.99 and 1.96 (1H, *J* = 18.4 Hz). The ^13^C NMR spectral data were related to friedelane-3,15-dione (**4**)^13^ and 28-hydroxyfriedelan-3-one (**6**),^14^ suggesting that compound **1** has a friedelane skeleton with carbonyl groups at C-3 (δ_C_ 213.1) and C-15 (δ_C_ 213.9), and a hydroxyl group at C-28 (δ_C_ 67.6). The HSQC spectrum showed correlations of C-23 (δ_C_ 6.8) with the doublet at δ_H_ 0.89; of C-28 (δ_C_ 67.6) with the two doublets at δ_H_ 3.69 and 3.79, and of C-16 (δ_C_ 47.6) with the two doublets at δ_H_ 2.99 and 1.96. The HMBC spectrum showed correlations of the doublet at δ_H_ 0.89 (H-23) with C-3 (δ_C_ 213.1), of the two doublets at δ_H_ 2.99 and 1.96 (H-16) with carbons C-15 (δ_C_ 213.9), C-17 (δ_C_ 39.3), C-18 (δ_C_ 40.1) and C-28 (δ_C_ 67.6) and of H-26 (δ_H_ 1.20) with C-15 (δ_C_ 213.9) (Figure 2). Besides that, the hydrogens H-28 (δ_H_ 3.69 e 3.79) correlated with carbons C-22 (δ_C_ 31.9), C-17 (δ_C_ 39.3), C-18 (δ_C_ 40.1) and C-16 (δ_C_ 47.6), in the HMBC spectrum. The COSY spectrum displayed correlations between the two hydrogens H-16 (δ_H_ 2.99 and 1.96) and between the two hydrogens H-28 (δ_H_ 3.69 and 3.79). In addition, correlations were observed between H-4 (δ_H_ 2.30) and H-23 (δ_H_ 0.89), as well as between H-1 (δ_H_ 1.96) and H-2 (δ_H_ 2.39) (Figure 2). Last, the NOESY spectrum showed correlations between the hydrogens H-1β (δ_H_ 1.68) and H-24 (δ_H_ 0.75); H-8α (δ_H_ 1.87) and H-10α (δ_H_ 1.60–1.62); H-8 (δ_H_ 1.87) and H-27 (δ_H_ 0.99); H-18β (δ_H_ 1.67) and H-16β (δ_H_ 1.96), H-18 and H-25 (δ_H_ 0.91), H-18 and H-26 (δ_H_ 1.20); H-28 (δ_H_ 3.79) and H-30 (δ_H_ 0.99), among others (Figure 2). The absolute configuration of the carbons in the friedelane skeleton has already been determined by X-ray diffraction,^15^ and for biosynthetic reasons, all friedelane triterpenes are formed in the same way, with the same absolute configuration. Based on the NMR data, compound **1** was identified as a new friedelane, 28-hydroxyfriedelane-3,15-dione (**1**).

The known compounds were identified from their respective ^13^C NMR data, compared with data from the literature, as friedelan-3-one (**2**),^14^ friedelan-3β-ol (**3**),^16^ friedelane-3,15-dione (**4**),^13^ 15α-hydroxyfriedelan-3-one (**5**),^13^ 28-hydroxyfriedelan-3-one (**6**),^17^ 30-hydroxyfriedelan-3-one (**7**)^18^ and 29-hydroxyfriedelan-3-one (**8**).^16^

### Antiviral Assay

The antiviral activity of compounds **1**–**8** and the hexane (**EH**), chloroform (**EC**) and ethyl acetate (**EAE**) extracts was evaluated against the murine coronavirus MHV-3 (Table 2). L929 cells were initially used to establish the CC_50_ values and the SI values were determined for the tested compounds. Ribavirin (10 μM) was used as the positive control. **EH** and **EAE** showed low antiviral activity (186 ± 16 μg mL^–1^ and 167 ± 15 μg mL^–1^, respectively). Among the tested triterpenes, compounds **1** and **6** showed the best results (69 ± 6 and 2.9 ± 0.3 μM, SI > 1.5 and >34.4, respectively). Additionally, none of the compounds showed activity against the *S. aureus* or MRSA strains up to 100 μM or 200 μg/mL.

**Table 2:** Biological Evaluation of Compounds against MHV-3 in L929 Cells^a^

**compound**	**CC**_**50**_	**EC**_**50**_	**SI**
1	>100 μM	69 ± 6 μM	>1.5
2	>100 μM	NA^a^	
3	>100 μM	NA	
4	>100 μM	NA	
5	>100 μM	NA	
6	>100 μM	2.9 ± 0.3 μM	>34.4
7	>100 μM	NA	
8	>100 μM	NA	
EH	>200 μg/mL	186 ± 16 μg/mL	>1.1
EC	>200 μg/mL	NA	
EAE	>200 μg/mL	167 ± 15 μg/mL	>1.2
ribavirin	>100 μM	<10 μM	IS > 10

a NA: not active.

Both active triterpenes (**1** and **6**) have a hydroxyl group at C-28, and the monocarbonyl triterpene **6** showed a 23-times increase in activity compared to the new isolated triterpene **1**. Interestingly, compound **4**, a nonhydroxylated analog of compound **1**, showed no antiviral activity up to 100 μM. The other triterpenes that lack the hydroxyl group at C-28 were also inactive. These results suggest the presence of the hydroxyl group at C-28 is essential for the antiviral activity against MHV-3, and that the presence of a second carbonyl group reduces activity. Therefore, further testing with other hydroxylated triterpenes at C-28 is needed to establish a better structure–activity relationship and to investigate the mechanism behind the observed antiviral activity.

### Cytotoxic Assay

All tested compounds (**1**, **4**, **6** and **7**) showed high IC_50_ values, ranging from 350 to 623 μM against THP-1 cells (positive control Cytarabine, IC_50_ of 41 ± 8 μM), and from 259 to 593 μM against K-562 cells (positive control Imatinib, IC_50_ of 35 ± 4 μM). The compound **4** (friedelane-3,15-dione) exhibited the highest cytotoxic activity against THP-1 cells, with an IC_50_ value of 350 ± 43 μM. On the other hand, the most active compound against K-562 cells was the new triterpene **1** (28-hydroxyfriedelane-3,15-dione), with an IC_50_ of 259 ± 33 μM (Table 3).

**Table 3:** Cytotoxicity of the Compounds **1**, **4**, **6** and **7** against Leukemia Cell Lines

**IC**_**50**_**(μM) ± SD**^a^		
**compound**	**THP-1**	**K-562**
1	539 ± 43	259 ± 33
4	350 ± 43	593 ± 44
6	623 ± 12	318 ± 41
7	385 ± 21	361 ± 52
imatinib		35 ± 4
cytarabine	41 ± 8	

a The IC_50_ values were presented as mean ± standard deviation (SD) of two independent experiments.

## Conclusions

The phytochemical study of *Salacia grandifolia* leaves led to the isolation of eight pentacyclic triterpenes with a friedelane skeleton. To the best of our knowledge, the NMR data (1D and 2D) of 28-hydroxyfriedelane-3,15-dione (**1**) is herein reported for the first time. All tested compounds showed low cytotoxic activity against THP-1 and K-562 leukemia cells. Compound **6** (28-hydroxyfriedelan-3-one) exhibited higher antiviral activity compared to **1** against a mouse coronavirus, and both essentially did not reduce the viability of L929 cells.

## Supporting Information Available

The IR and NMR spectra of compounds **1**-**8** and the HR-IT-TOF-MS analysis of compound **1,** are available in the Supporting Information (Figures S1–S28), as well as the NMR data comparison of compound **1** (Table S1). (PDF)
