# Supplementary material for: Isolation and In Vitro Biological Evaluation of Triterpenes from Salacia grandifolia Leaves
Source: ACS Omega. 2024 Jul 11;9(29):32153–8. doi: 10.1021/acsomega.4c04360 (PMC11270730; doi:10.1021/acsomega.4c04360)
Supplement: Supplementary file 1 — ao4c04360_si_001.pdf [file ao4c04360_si_001.pdf]

Supplementary Information

Isolation and *In Vitro* Biological Evaluation of Triterpenes From *Salacia grandifolia* Leaves

Leila Renan Oliveira[a], Mateus Sá Magalhães Serafim[b], Diego Lanza Dias[b], Túlio Resende Freitas[c], Jonatas Santos Abrahao[b], Guilherme de Medeiros Antar[d], Bruno E. F. Mota[c], Adriano de Paula Sabino[c], Lucienir Pains Duarte[a], Diogo Montes Vidal[a], Grasiely Faria de Sousa[a*]

[a]*Departamento de Química, Universidade Federal de Minas Gerais, 31270-901 Belo Horizonte-MG, Brasil*

[b]*Departamento de Microbiologia, Instituto de Ciências Biológicas, Universidade Federal de Minas Gerais, 31270-901 Belo Horizonte-MG, Brasil*

[c]*Departamento de Análises Clínicas e Toxicológicas, Faculdade de Farmácia, Universidade Federal de Minas Gerais, 31270-901 Belo Horizonte-MG, Brasil*

[d]*Departamento de Ciências Agrárias e Biológicas, Universidade Federal do Espírito Santo - Campus São Mateus, 29932-540 São Mateus-ES, Brasil*

*grasielysousa@ufmg.br

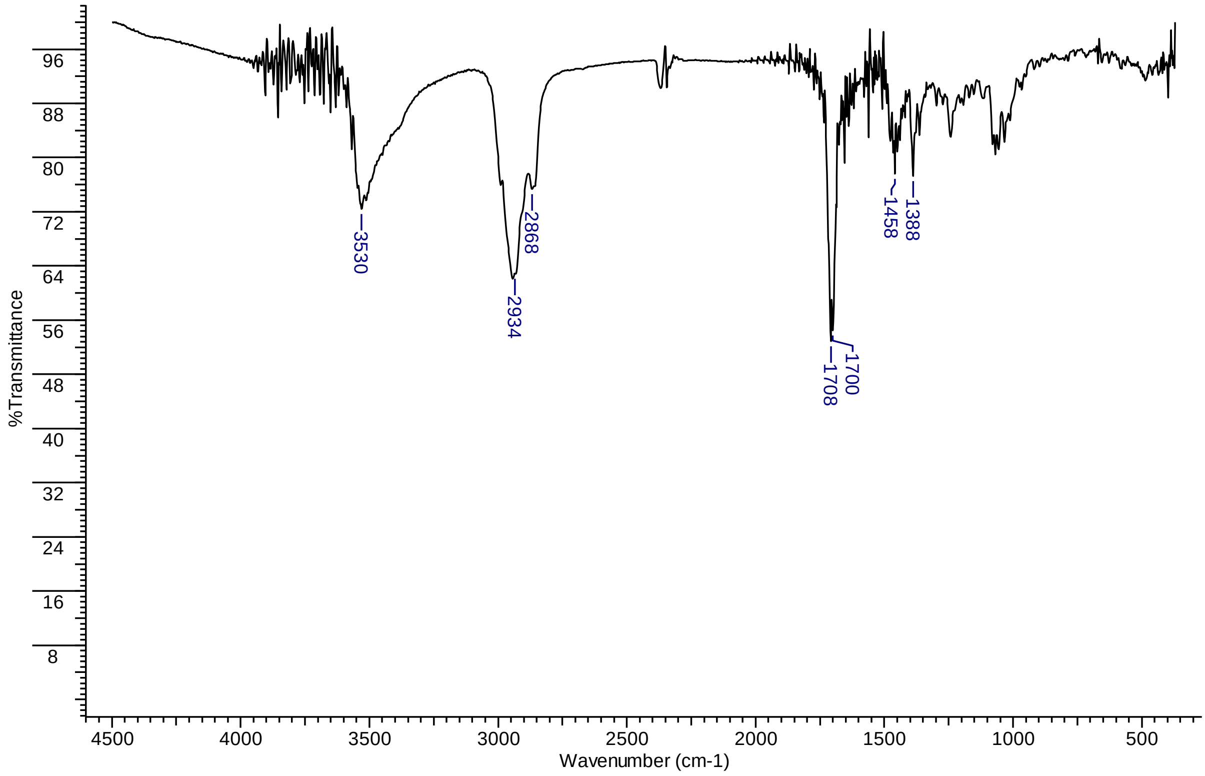

Figure S1. FTIR (KBr) spectrum of compound **1**.

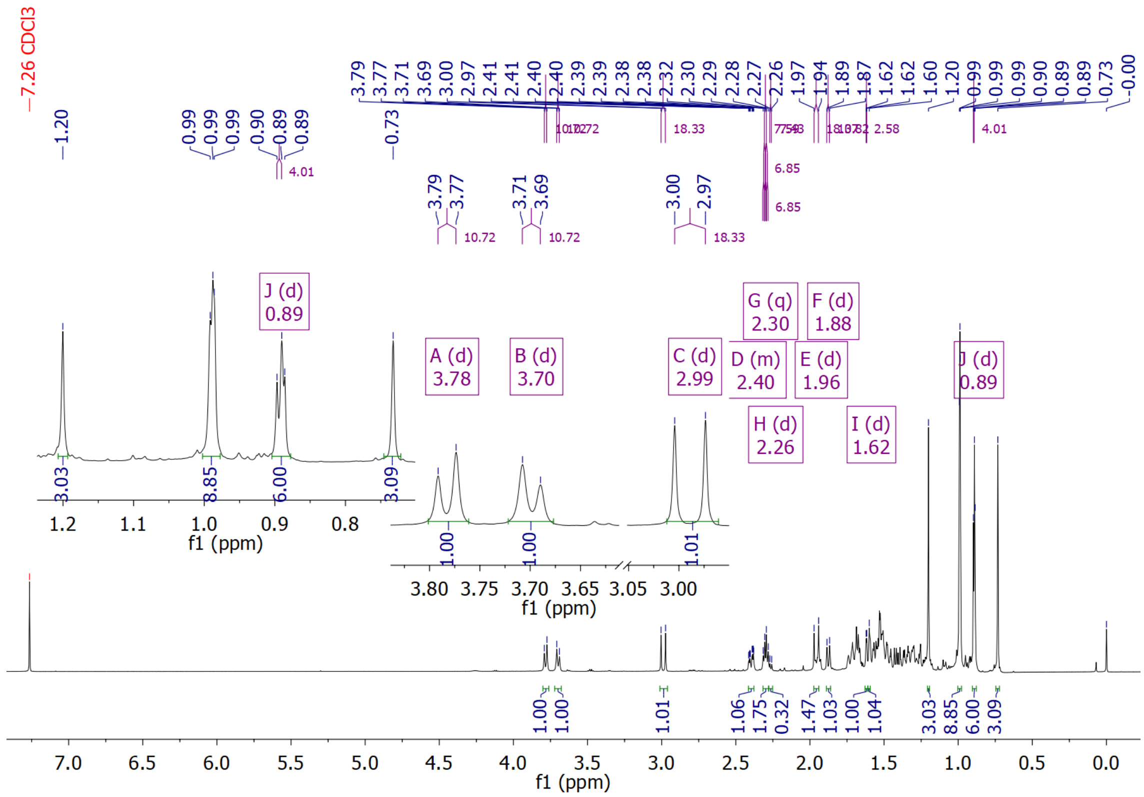

Figure S2. ^{1}H NMR spectrum (600 MHz, $CDCl_3$) of compound **1**.

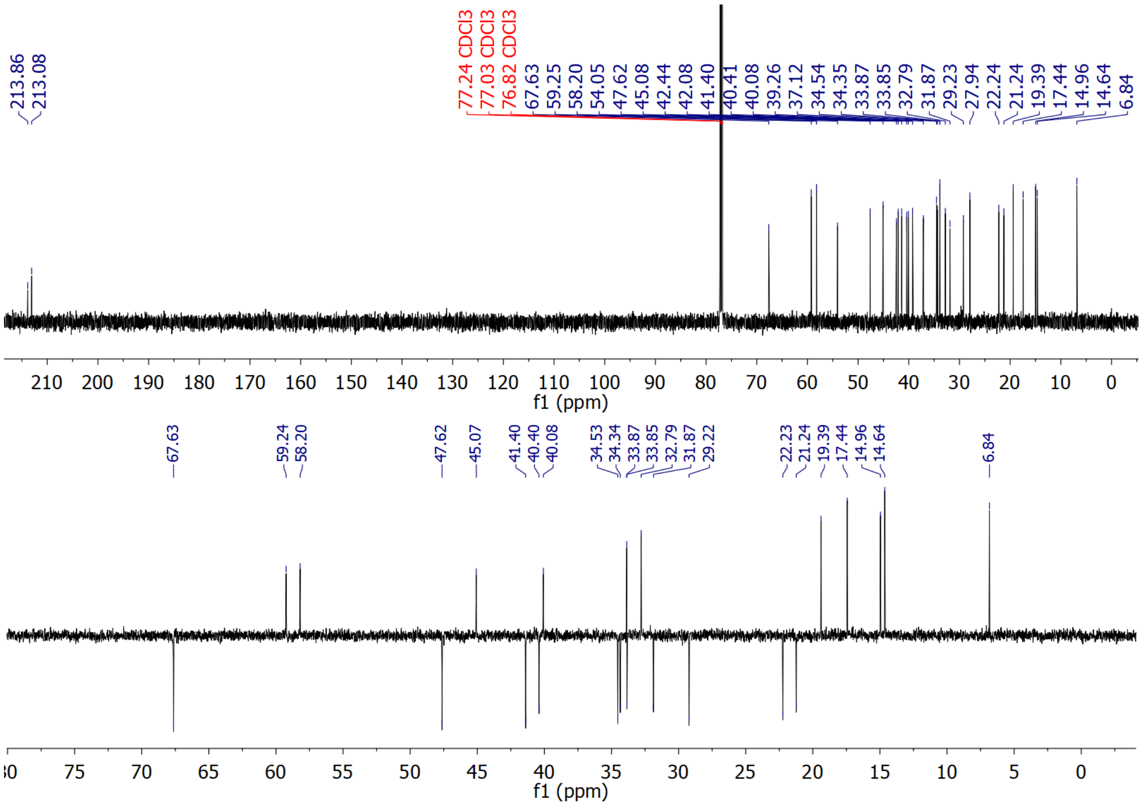

Figure S3. ^{13}C NMR and DEPT-135 spectra (150 MHz, $CDCl_3$) of compound **1**.

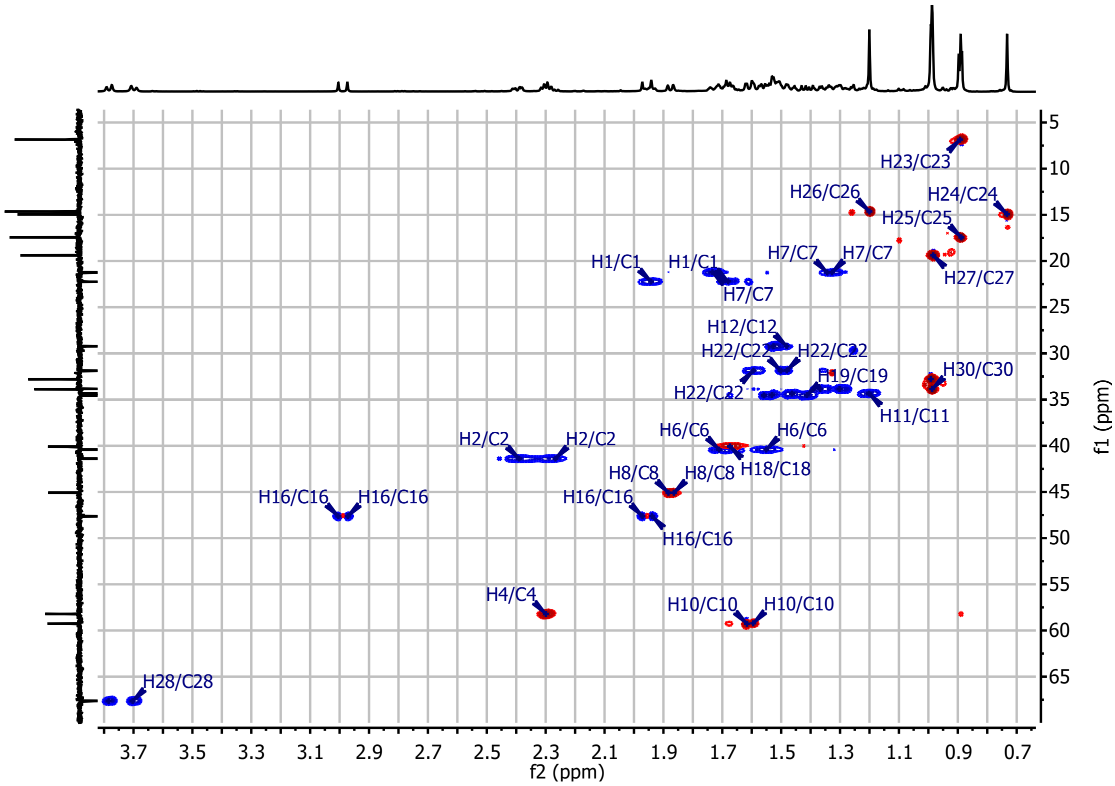

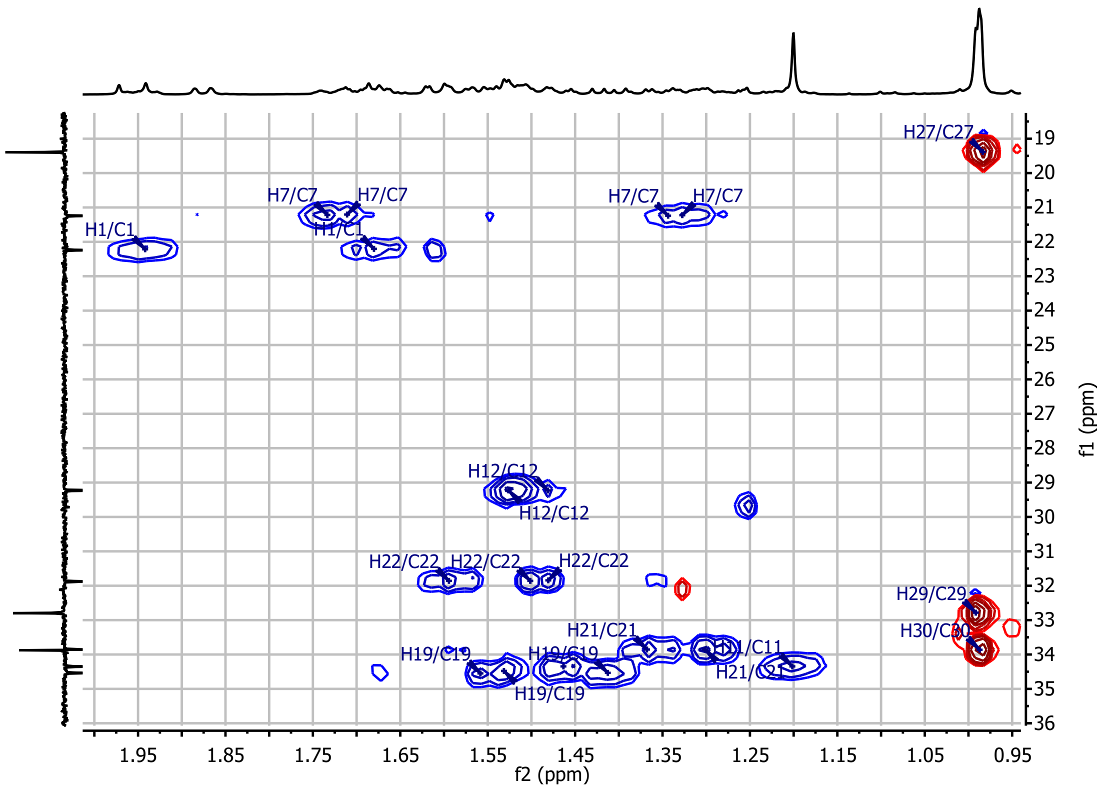

Figure S4. HSQC and expanded HSQC spectrum (600 MHz, $CDCl_3$) of compound **1** in the region between δ_H 2.0 to 0.8 ppm.

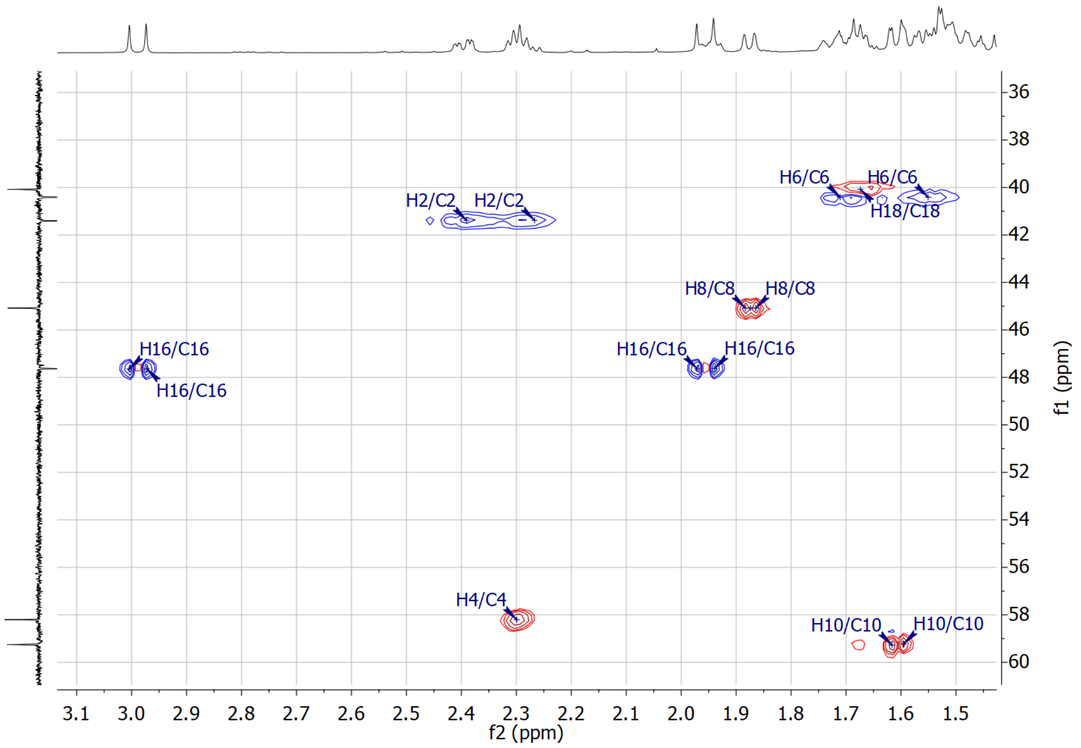

Figure S5. Expanded HSQC spectrum (600 MHz, $CDCl_3$) of compound **1** in the region between δ_H 3.1 to 1.5 ppm.

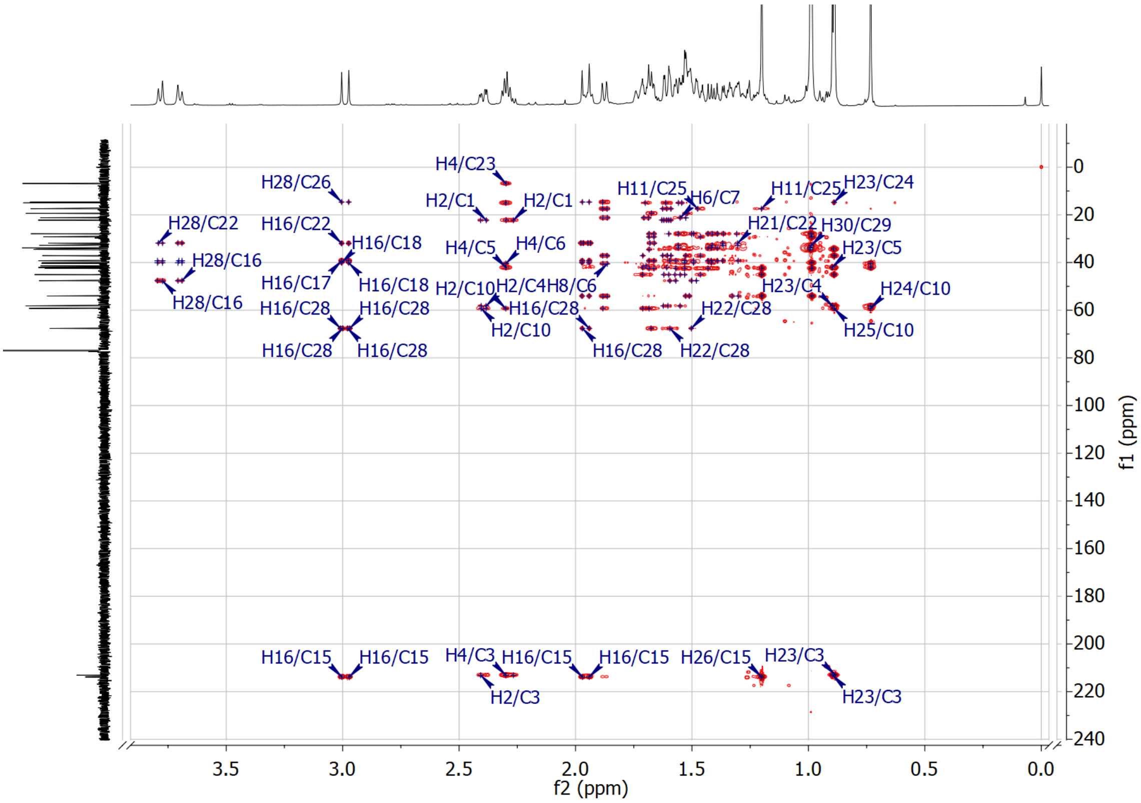

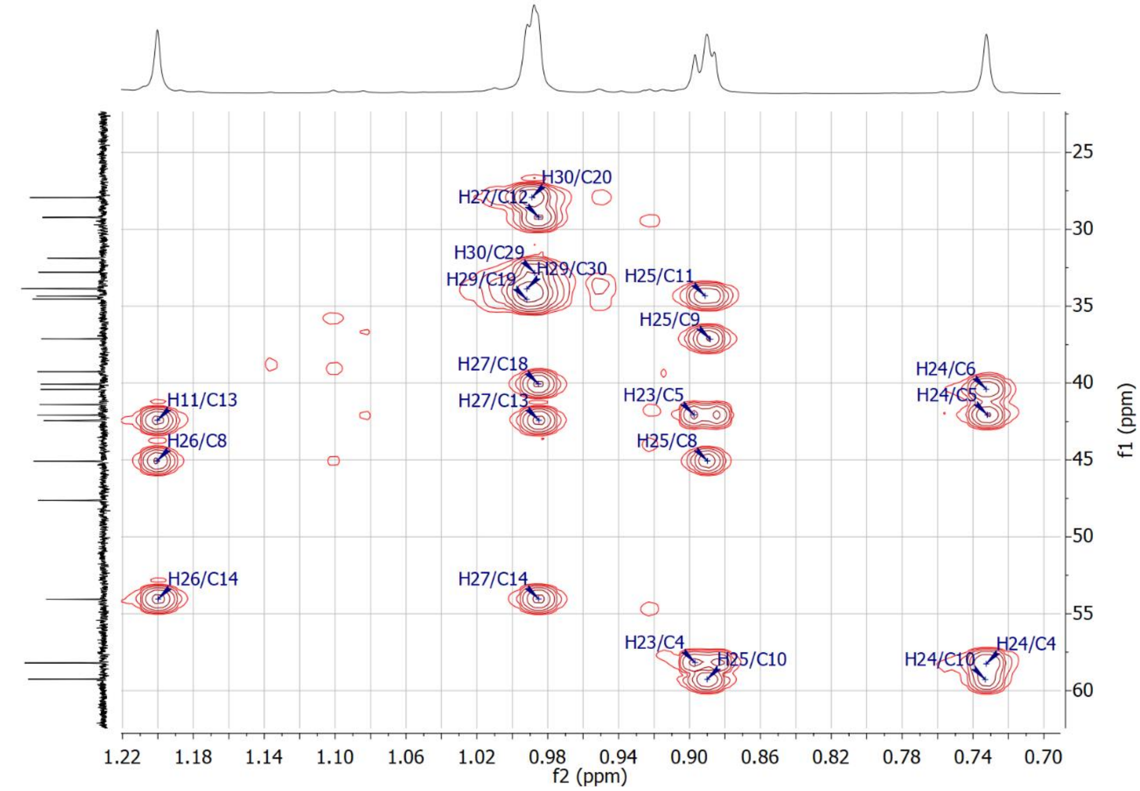

Figure S6. HMBC and expanded HMBC spectrum (600 MHz, $CDCl_3$) of compound **1** in the region between δ_H 1.22 to 0.70 ppm.

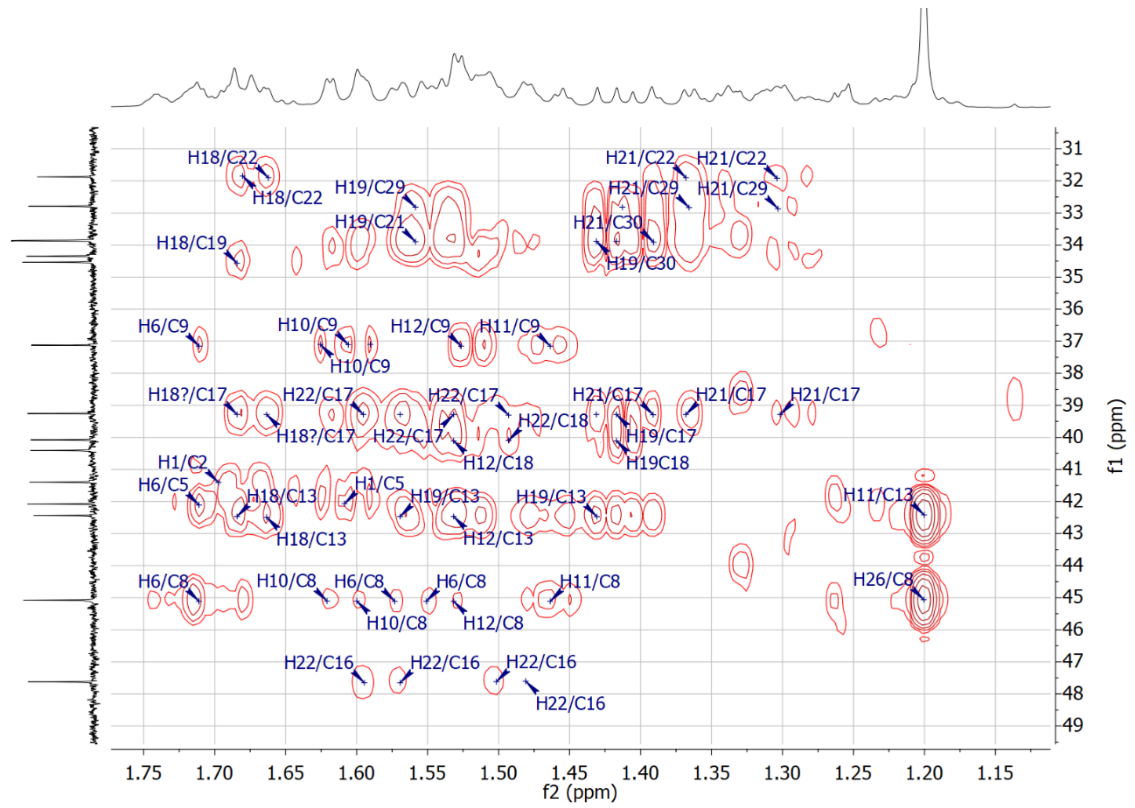

Figure S7. Expanded HMBC spectrum (600 MHz, $CDCl_3$) of compound **1** in the region between δ_H 1.75 to 1.15 ppm.

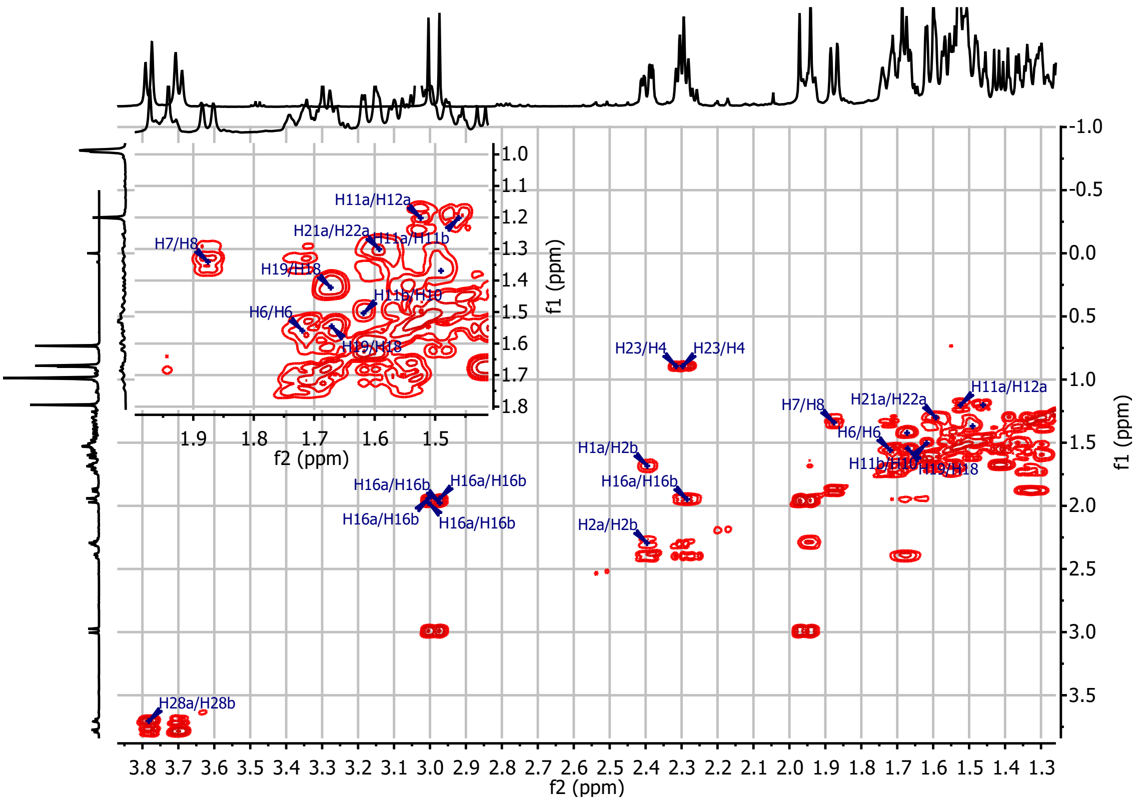

Figure S8. COSY and expanded COSY spectrum (600 MHz, $CDCl_3$) of compound **1** in the region between δ_H 1.90 to 1.40 ppm.

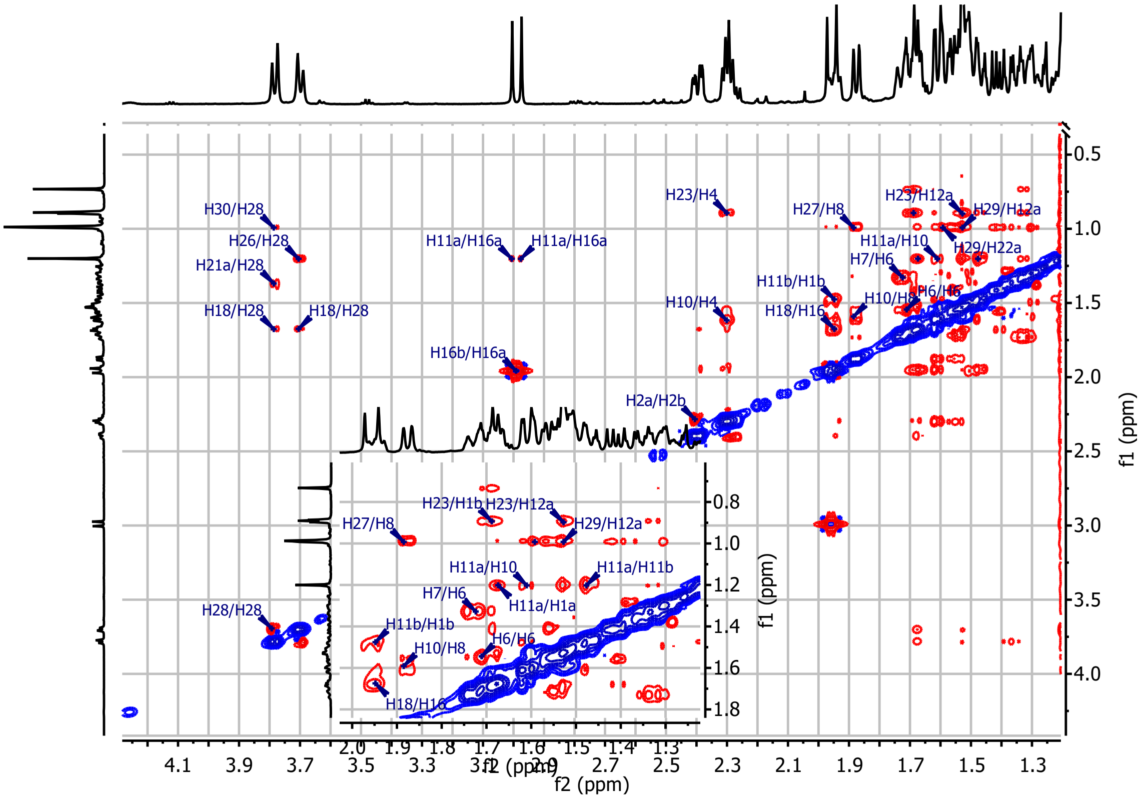

Figure S9. NOESY and expanded NOESY spectrum (600 MHz, $CDCl_3$) of compound **1** in the region between δ_H 1.80 to 1.40.

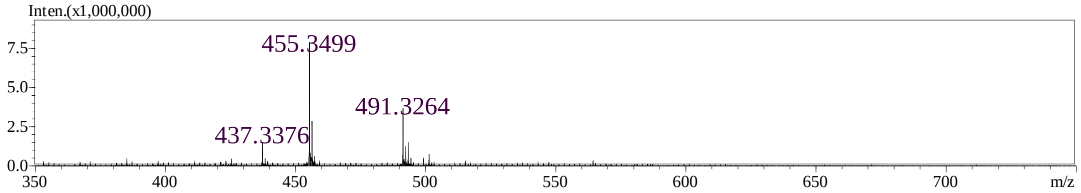

Figure S10. Mass spectrum (HR-IT-TOF-MS) of compound **1**.

Table S1. NMR (600 MHz, $CDCl_3$) data of compound **1**.

**Atom**	**Type**	**δ_C 1**	**δ_H 1**	**HMBC H→C**	**COSY**	**NOESY**	**δ_C friedelan-3,15-dione**[1]	**δ_C 28-hydroxyfrie-delan-3-one**[2]
**1**	CH_2	22.2	1.68β	2, 5, 10	2β	2β, 24, 23	22.3	22.1
			1.96α	-	-	11α		
**2**	CH_2	41.4	2.27α	1	1β	1α, 2β	41.4	41.3
			2.39β	1, 3, 4, 10	1α, 2α	2α		
**3**	C	213.1	-	-	-	-	213.1	213.6
**4**	CH	58.2	2.30α	1, 3, 5, 6, 10, 23, 24	23	10α, 23	58.2	57.8
**5**	C	42.1	-	-	-	-	42.0	41.9
**6**	CH_2	40.4	1.55	4, 7, 8, 24	6	6	40.5	41.0
			1.71	5, 7, 8, 9, 10, 24	7, 6	6, 7		
**7**	CH_2	21.2	1.35	14	-	6	21.3	18.1
			1.75	-	6	-		
**8**	CH	45.1	1.87α	6, 7, 9, 10, 14, 25, 26	7	10, 27	45.3	52.2
**9**	C	37.1	-	-	-	-	37.2	37.3
**10**	CH	59.3	1.60-1.62α	1, 4, 8, 9, 24, 25	-	4α, 11α	59.3	59.1
**11**	CH_2	34.4	1.20α	8, 13, 25	11β, 12α	1α, 10α, 16α	34.4	35.3
			1.48β	8, 9, 12, 13, 25	11α	-		
**12**	CH_2	29.2	1.48β	-	-	-	29.4	29.9
			1.53α	8, 9, 13, 14, 18, 27	11α	11α, 29		
**13**	C	42.4	-	-	-	-	42.4	39.1
**14**	C	54.1	-	-	-	-	54.2	38.0
**15**	C	213.9	-	-	-	-	214.9	31.3
**16**	CH_2	47.6	1.96β	15, 17, 18, 22, 26, 28	16α	16α, 18β	54.0	29.0
			2.99 α	15, 17, 18, 22, 26, 28	16β	11α, 16β		
**17**	C	39.3	-	-	-	-	33.5	35.1
**18**	CH	40.1	1.67β	12, 13, 17, 19, 20, 22, 27, 28	19	16β, 25, 26	44.0	39.2
**19**	CH_2	34.5	1.43	13, 17, 18, 20, 21, 29, 30	18	-	34.9	34.4
			1.55 - 1.58		18	-		
**20**	C	27.9	-	-	-	-	27.9	27.9
**21**	CH_2	33.9	1.32α	17, 20, 22, 29, 30	22α	29	33.8	31.4
			1.39β		22β	25		
**22**	CH_2	31.9	1.49β	16, 17, 18, 20, 28	21α	16β	38.6	33.2
			1.59α	16, 17, 18, 20	21β	8α, 29		
**23**	CH_3	6.8	0.89	3, 4, 5, 24	4	4, 1β	6.8	6.7
**24**	CH_3	15.0	0.75	4, 5, 6	-	1β	15.0	14.5
**25**	CH_3	17.4	0.91	8, 9, 10, 11	-	-	17.4	18.0
**26**	CH_3	14.6	1.20	8, 14, 15, 25	-	28	14.7	18.9
**27**	CH_3	19.4	0.99	12, 13, 14, 18	-	-	18.9	19.1
**28**	CH_2	67.6	3.69	16, 17, 18, 22	28	18β, 26β, 28	32.2	67.0
			3.79		28	18, 21β, 28, 30		
**29**	CH_3	32.8	0.99	19, 21, 30	-	12α, 21α, 22α	33.3	32.9
**30**	CH_3	33.9	0.99	20, 29	-	-	33.4	34.2

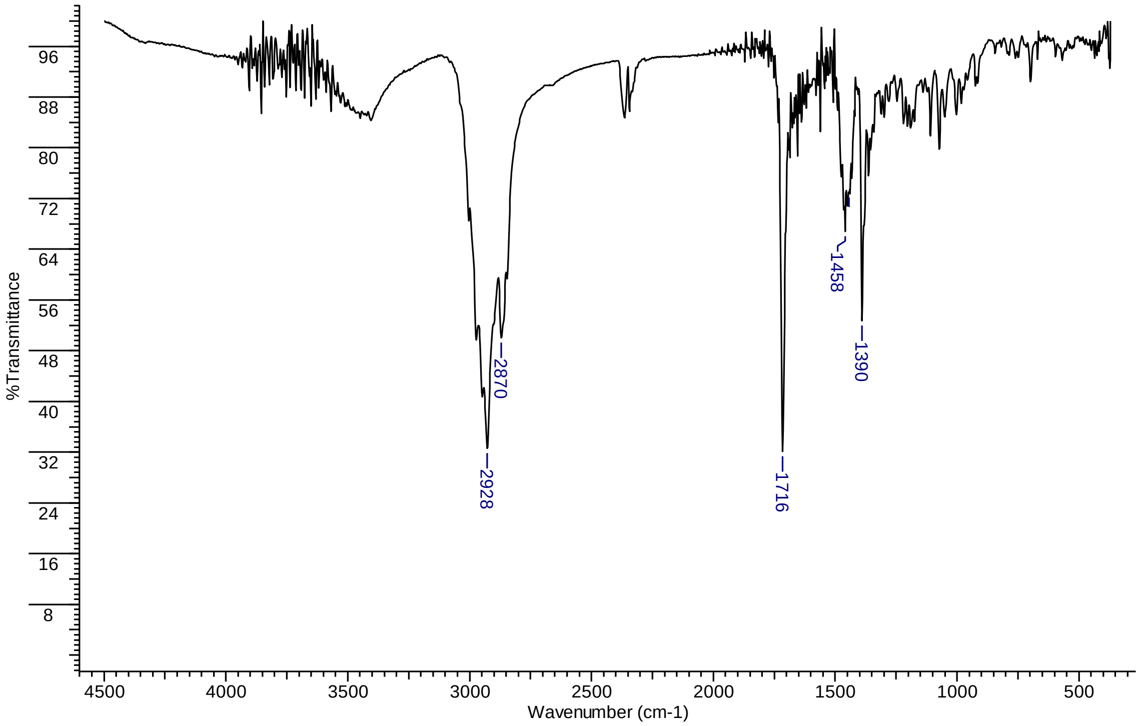

Figure S11. FTIR (KBr) spectrum of compound **2**.

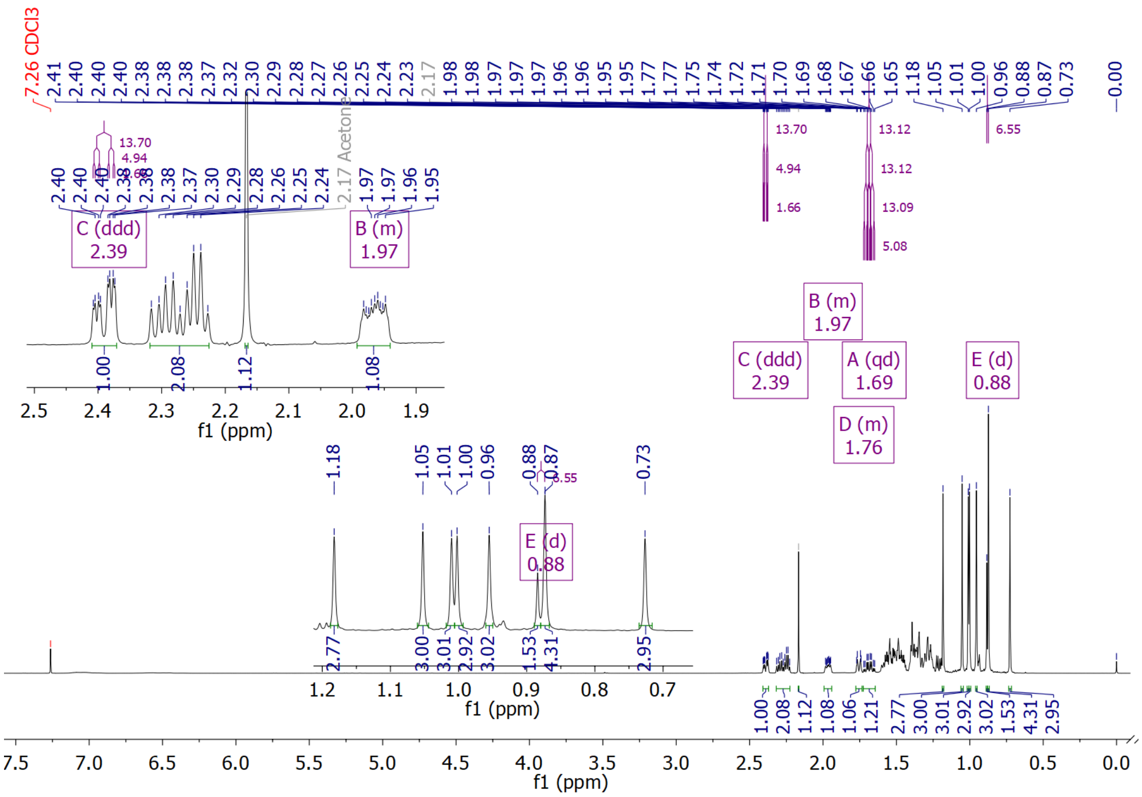

Figure S12. ^{1}H NMR spectrum (600 MHz, $CDCl_3$) of compound **2**.

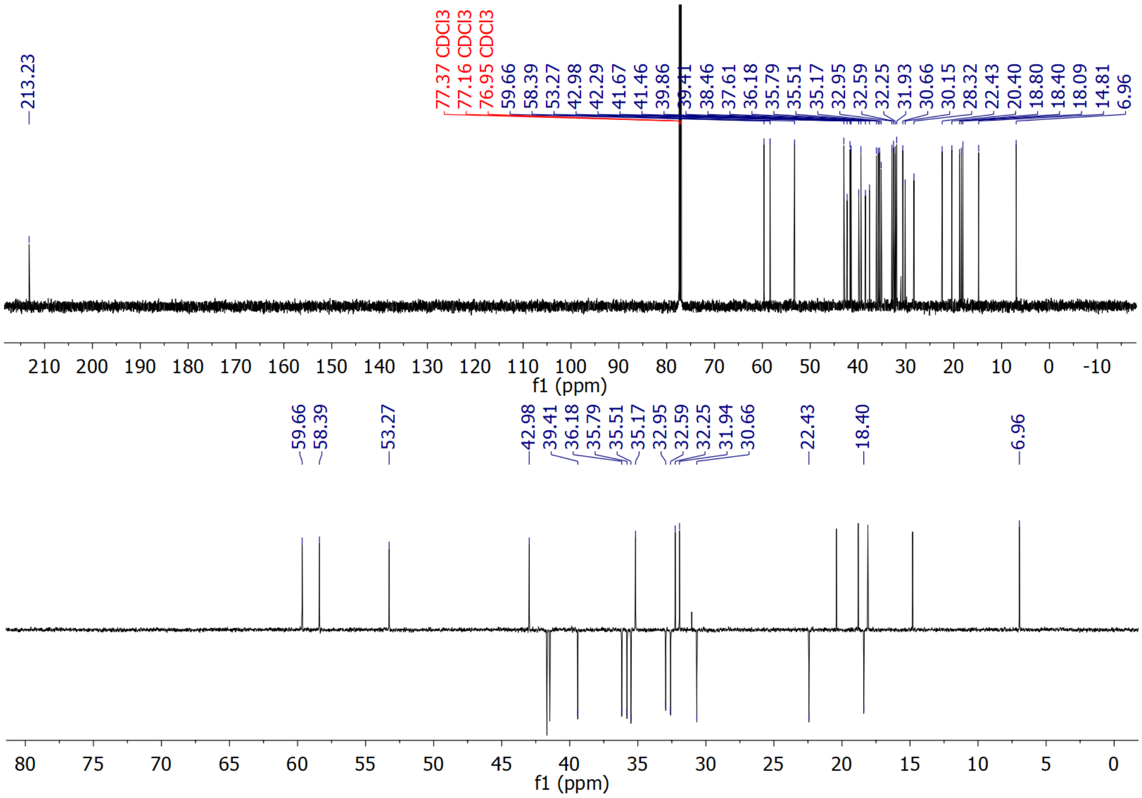

Figure S13. ^{13}C NMR and DEPT-135 spectra (150 MHz, $CDCl_3$) of compound **2**.

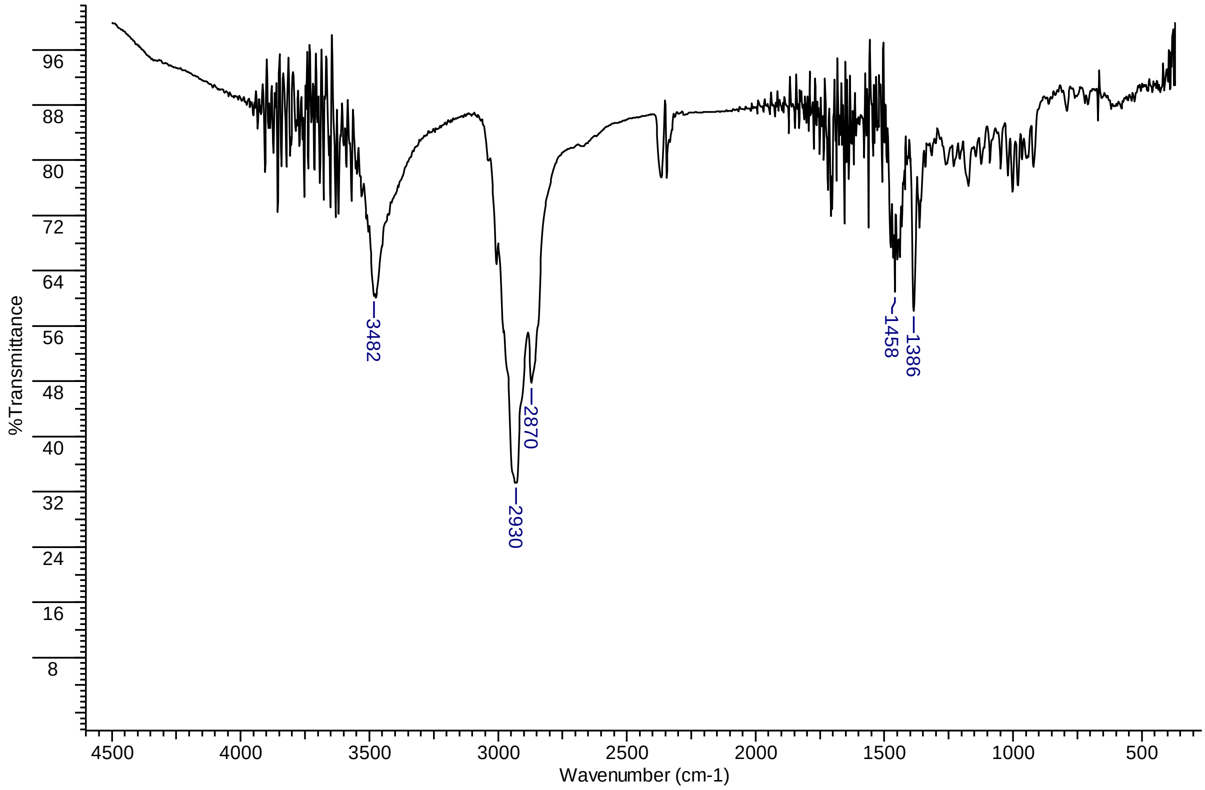

Figure S14. FTIR (KBr) spectrum of compound **3**.

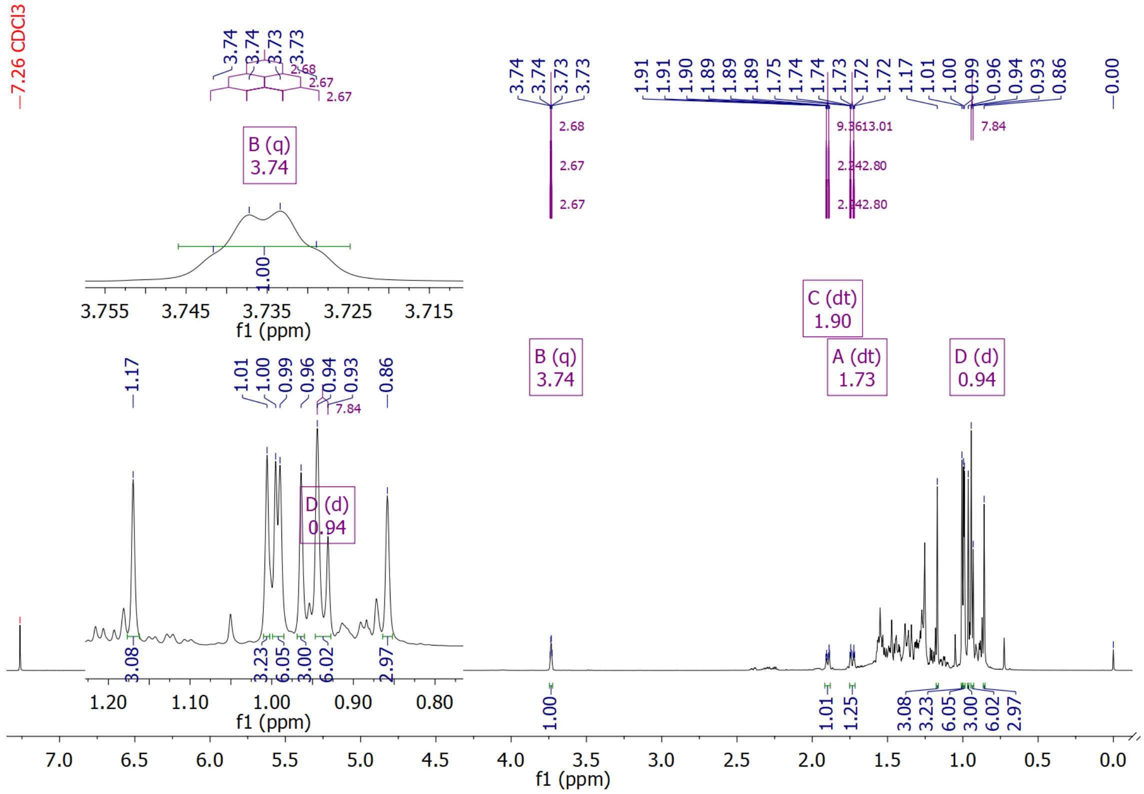

Figure S15. ^{1}H NMR spectrum (600 MHz, $CDCl_3$) of compound **3**.

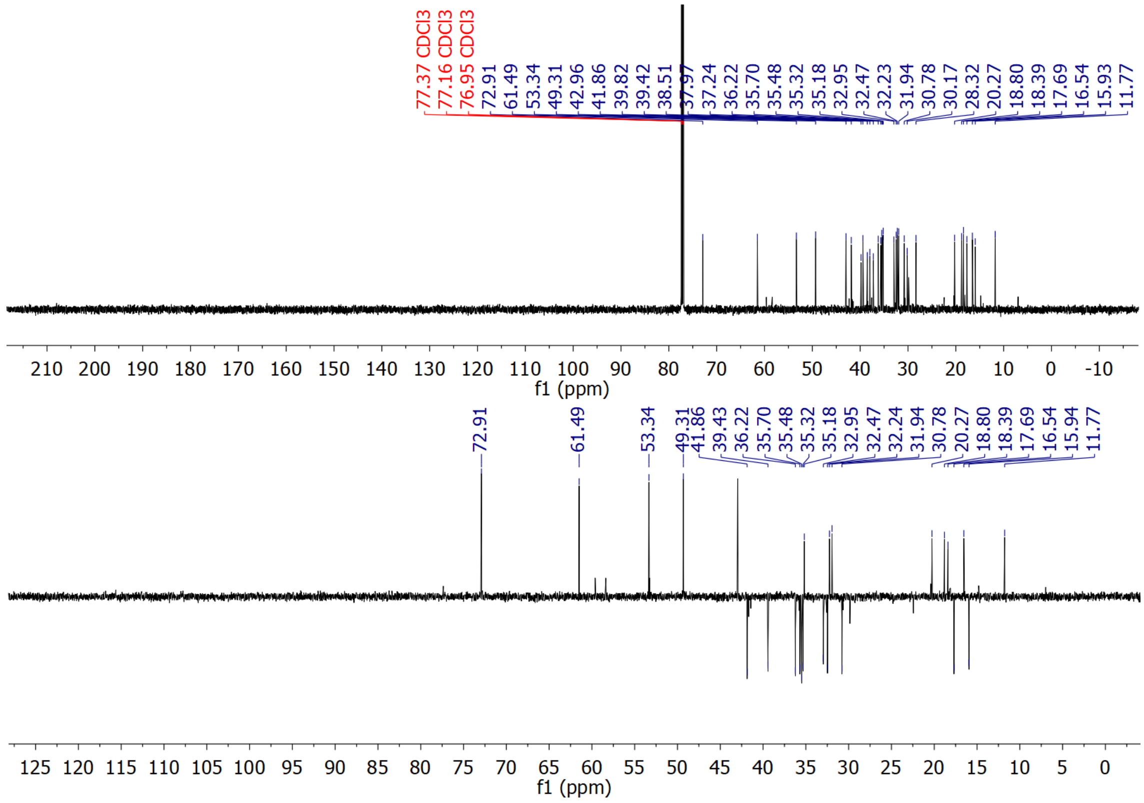

Figure S16. ^{13}C NMR and DEPT-135 spectra (150 MHz, $CDCl_3$) of compound **3**.

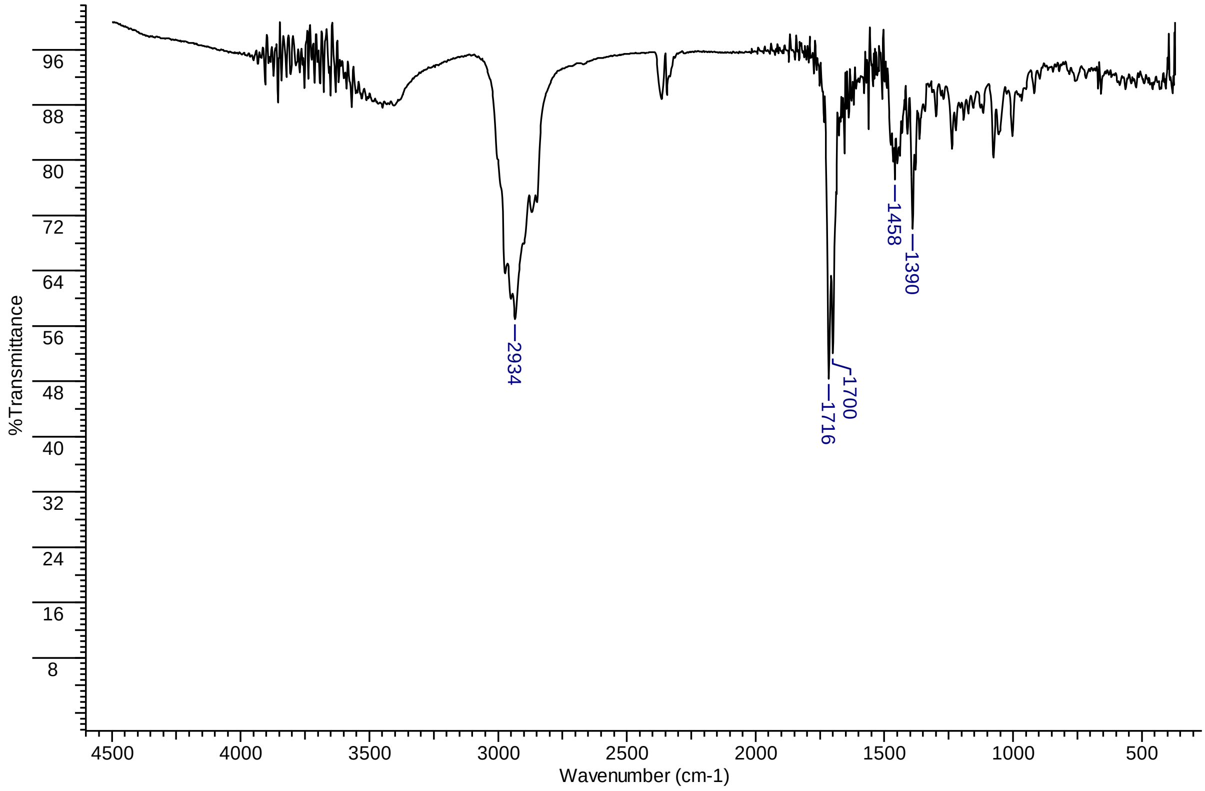

Figure S17. FTIR (KBr) spectrum of compound **4**.

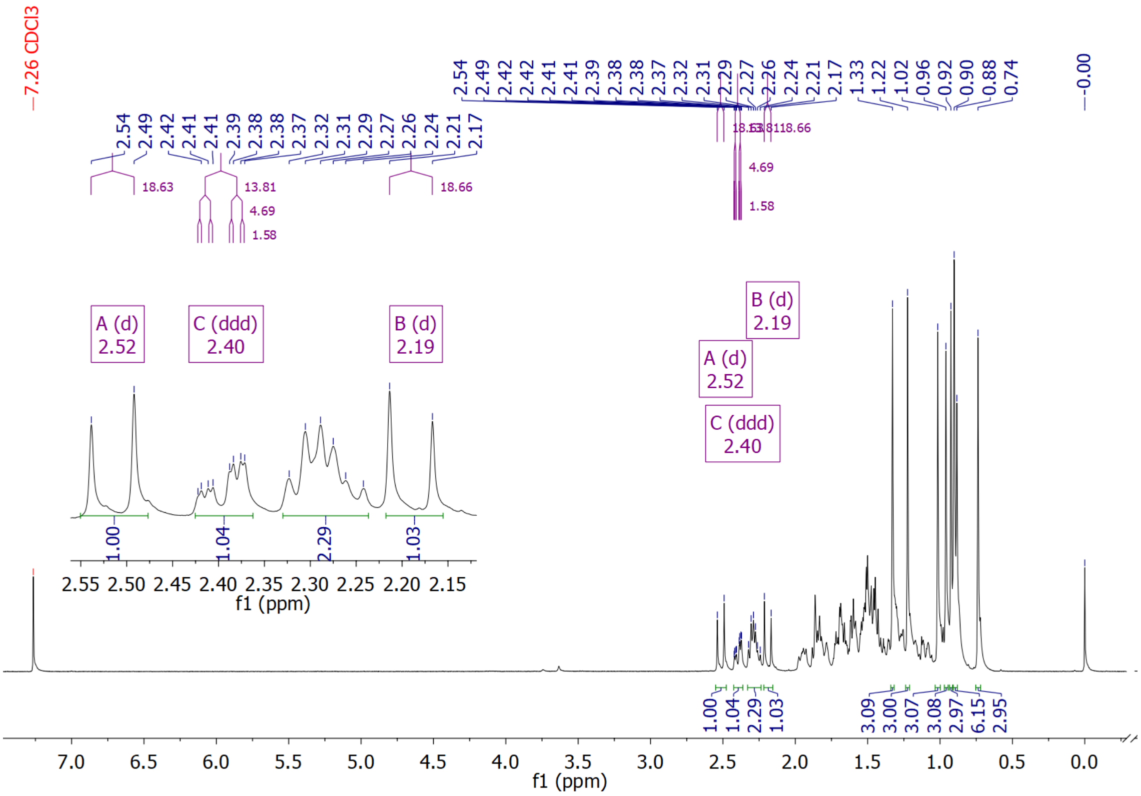

Figure S18. ^{1}H NMR spectrum (400 MHz, $CDCl_3$) of compound **4**.

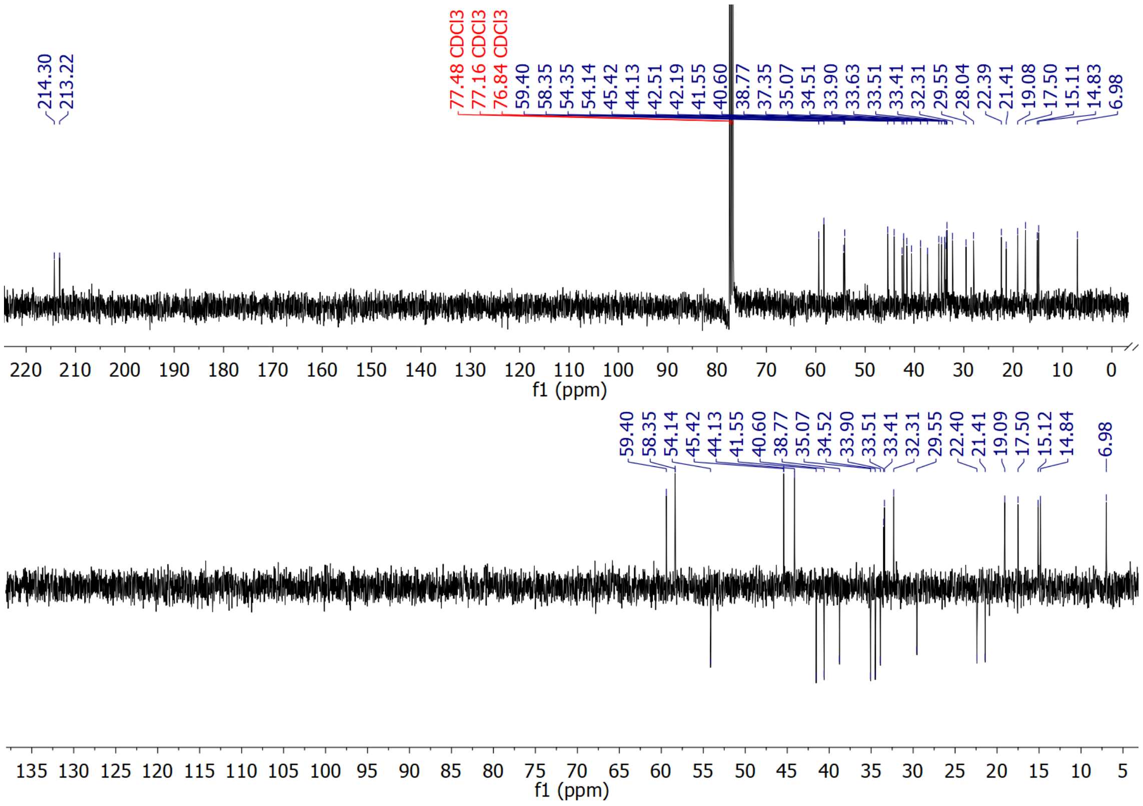

Figure S19. ^{13}C NMR and DEPT-135 spectra (100 MHz, $CDCl_3$) of compound **4**.

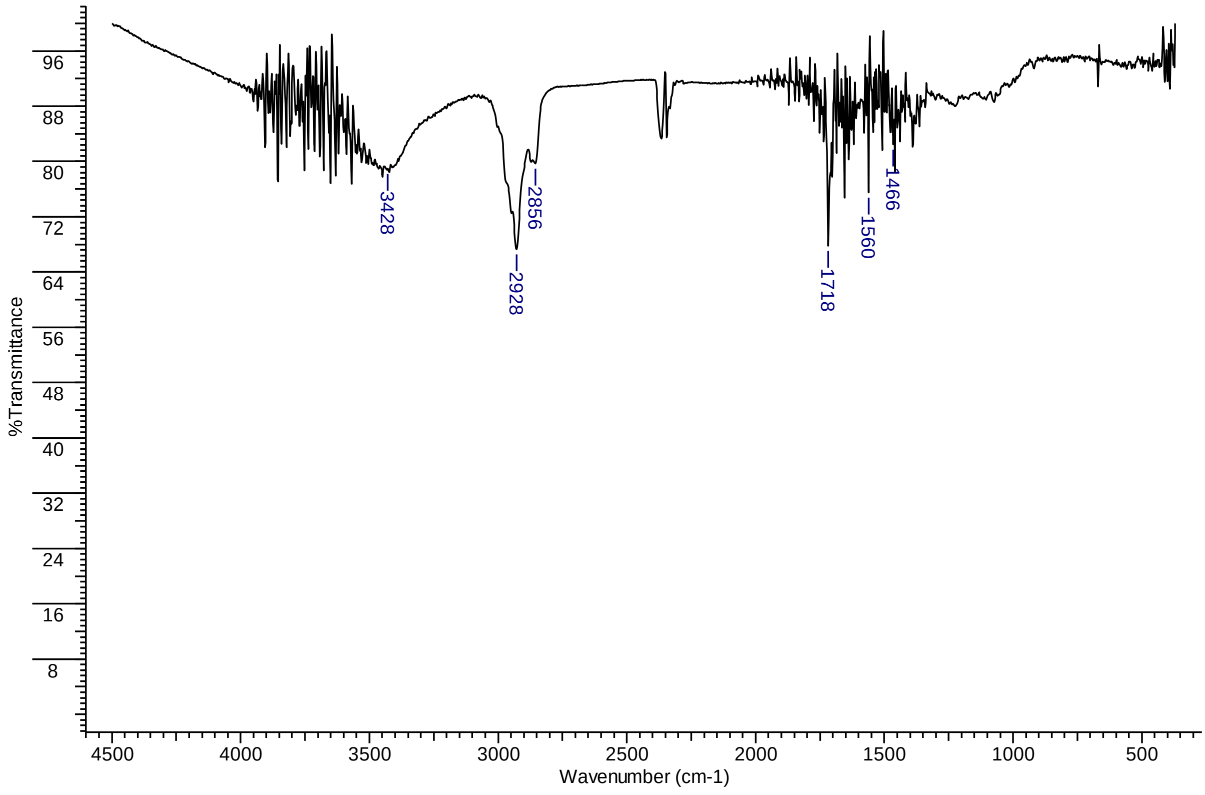

Figure S20. FTIR (KBr) spectrum of compound **5**.

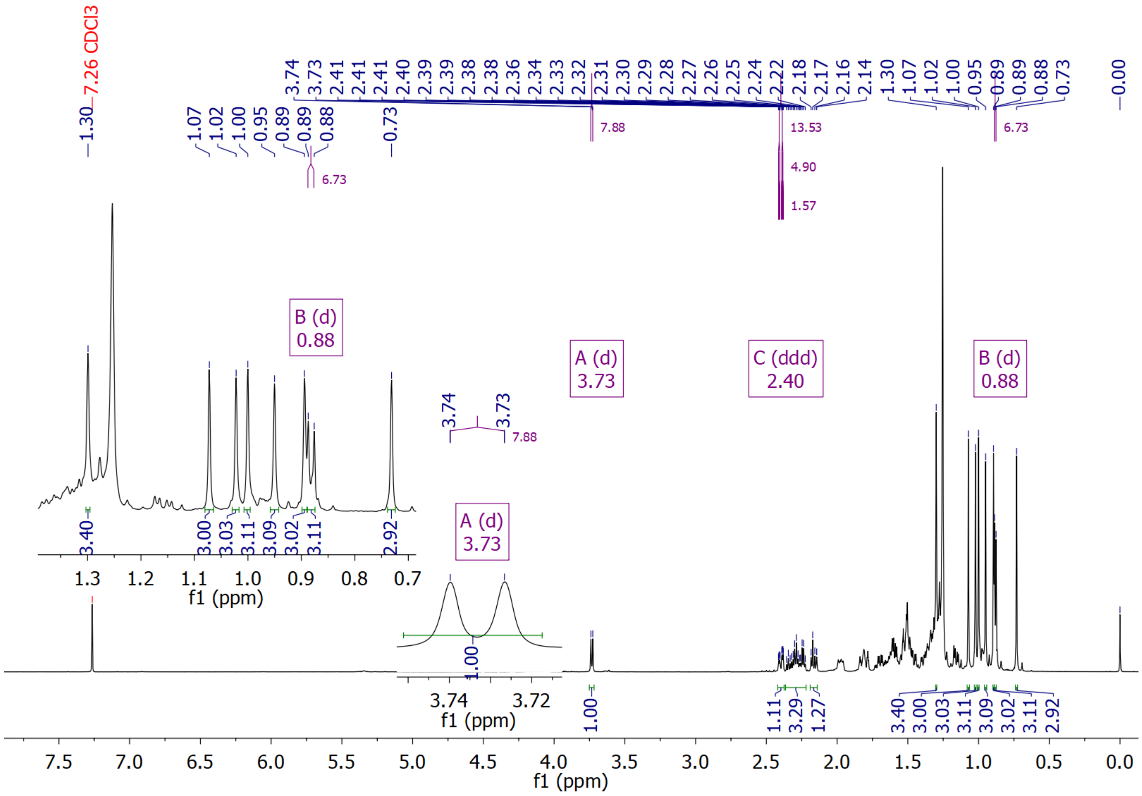

Figure S21. ^{1}H NMR spectrum (400 MHz, $CDCl_3$) of compound **5**.

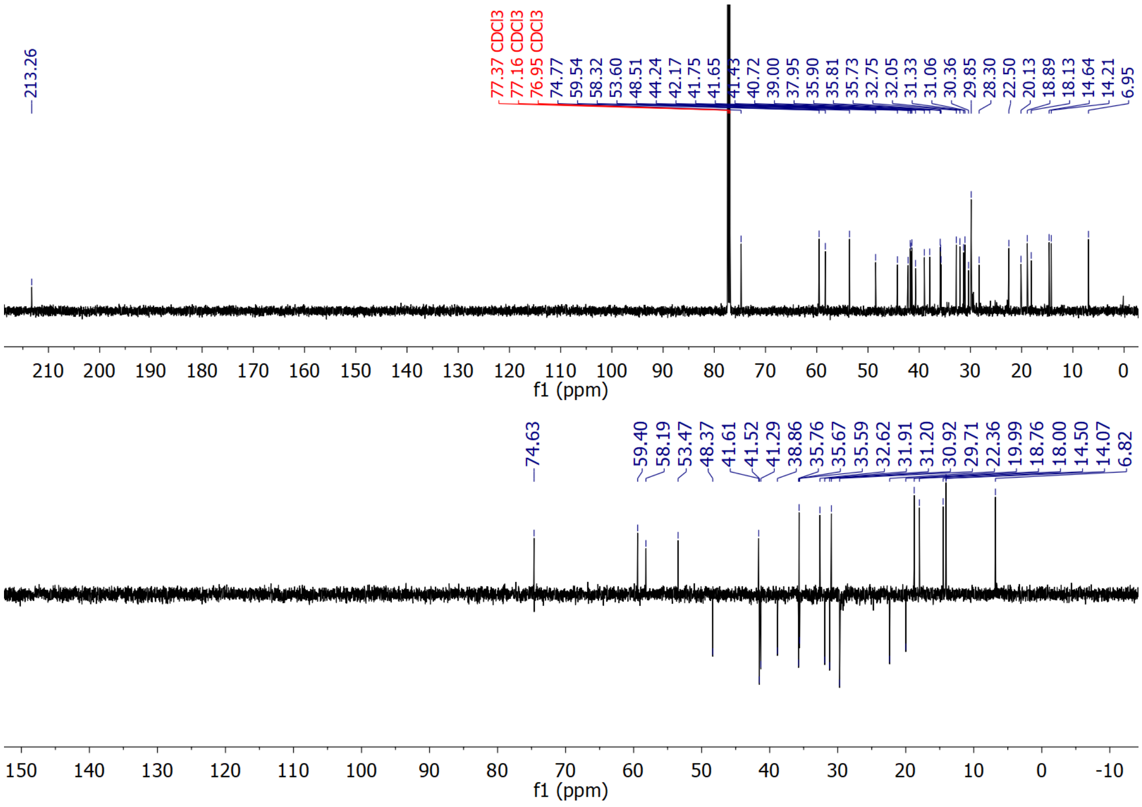

Figure S22. ^{13}C NMR and DEPT-135 spectra (100 MHz, $CDCl_3$) of compound **5**.

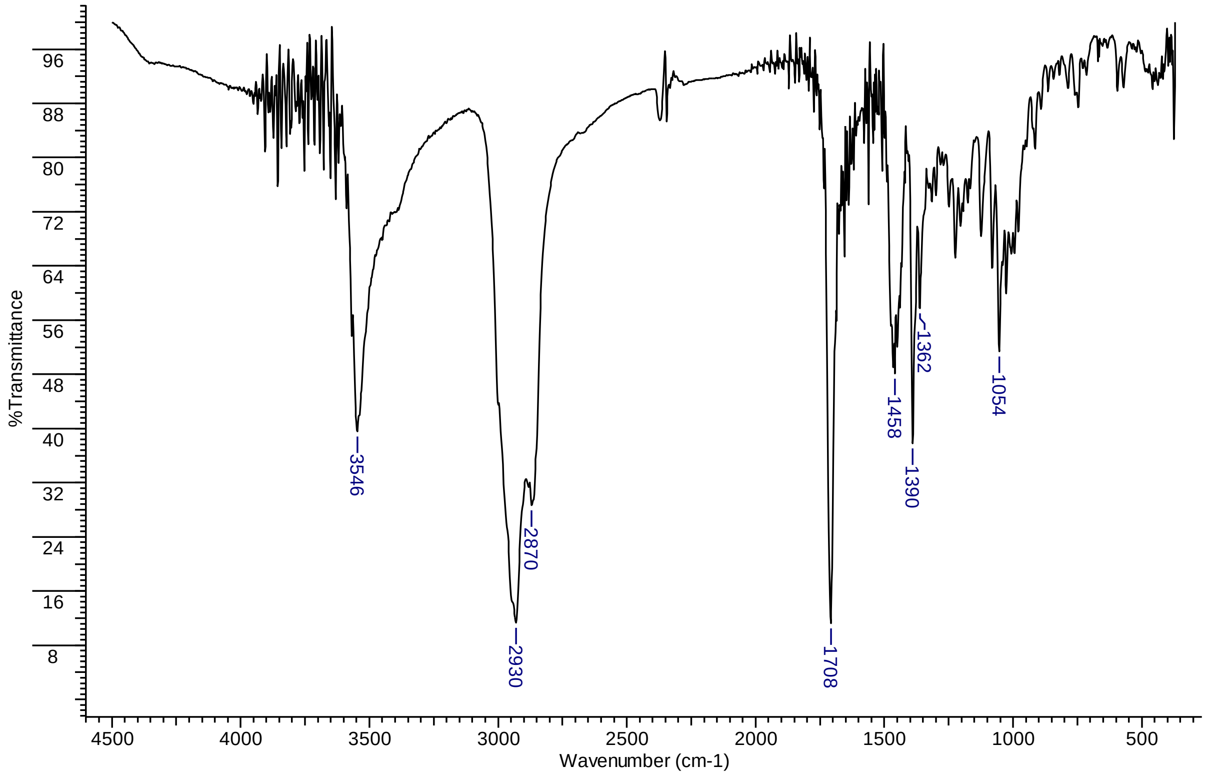

Figure S23. FTIR (KBr) spectrum of compound **6**.

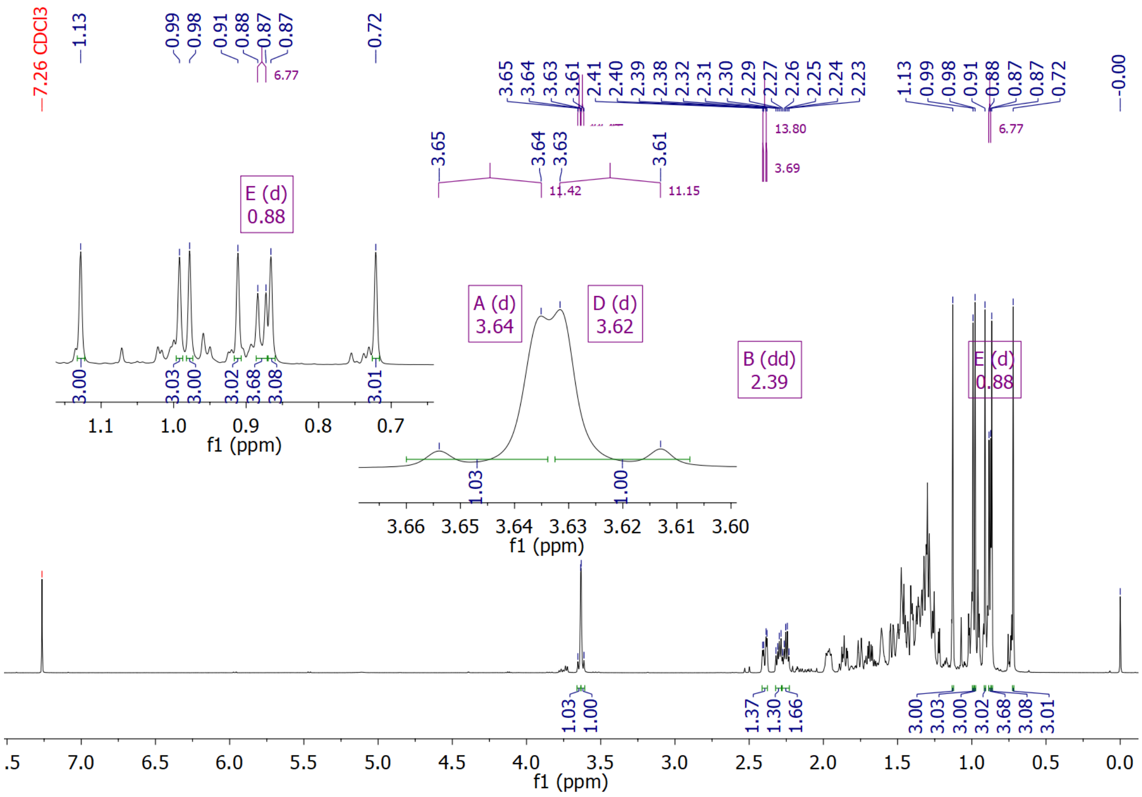

Figure S24. ^{1}H NMR spectrum (600 MHz, $CDCl_3$) of compound **6**.

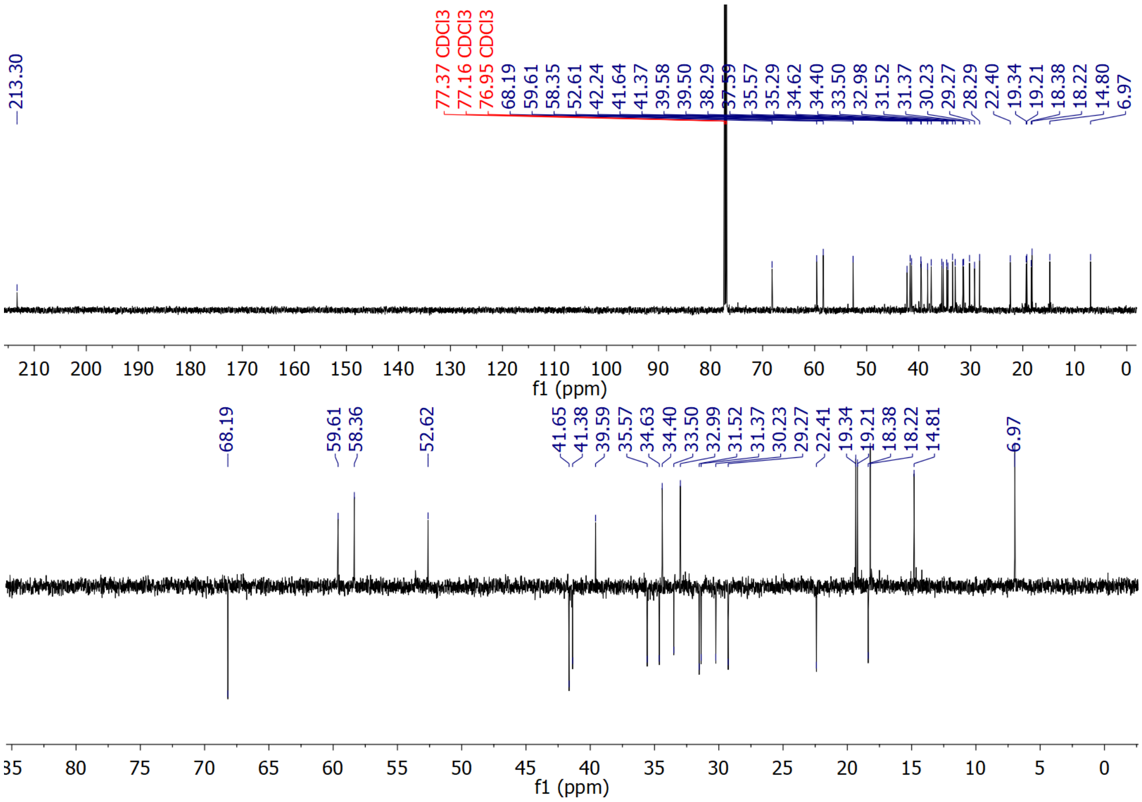

Figure S25. ^{13}C NMR and DEPT-135 spectra (150 MHz, $CDCl_3$) of compound **6**.

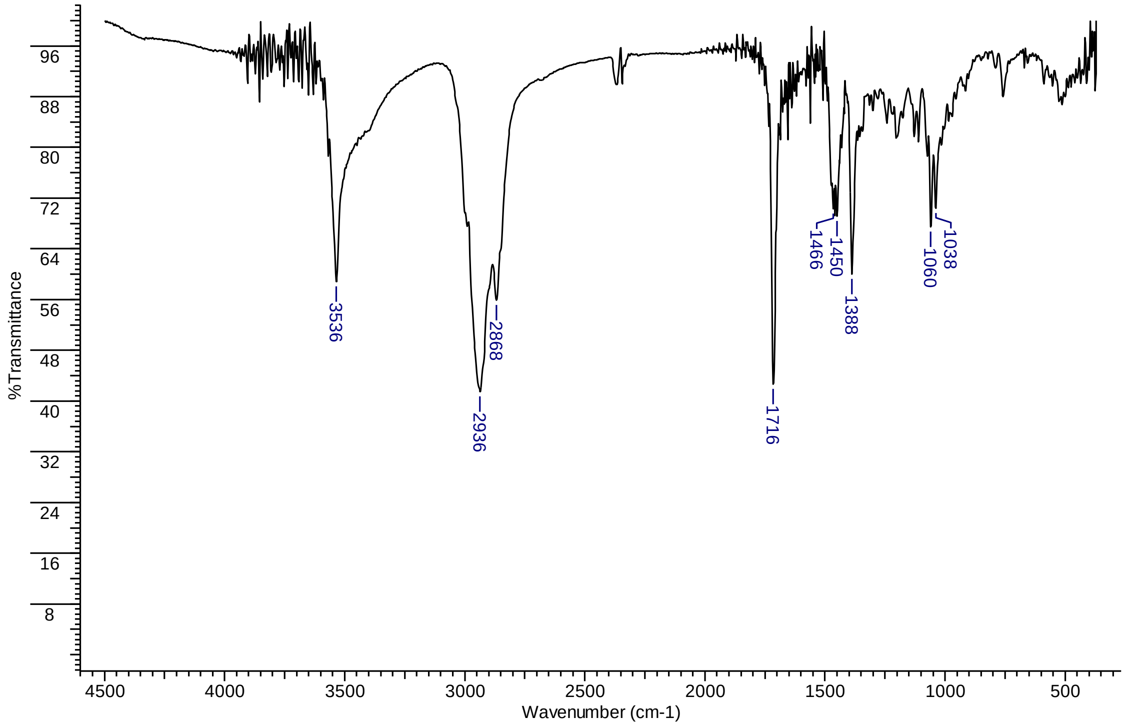

Figure S26. FTIR (KBr) spectrum of compound **7**.

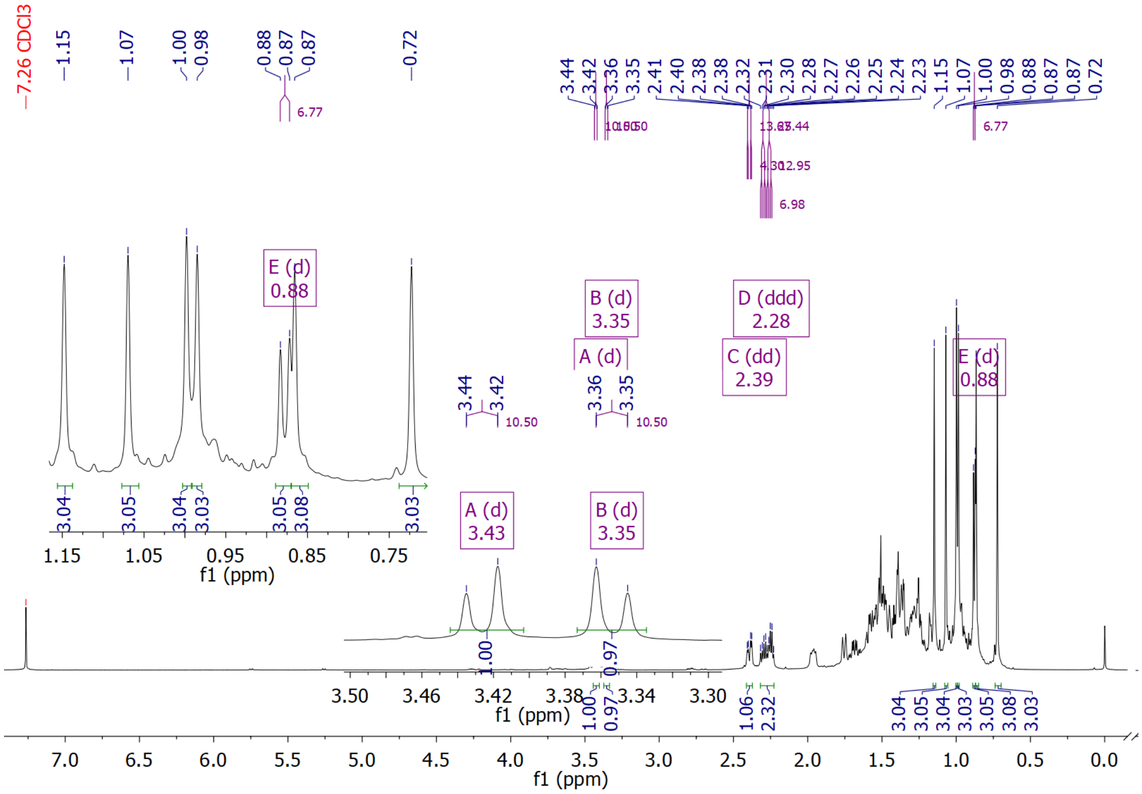

Figure S27. ^{1}H NMR spectrum (600 MHz, $CDCl_3$) of compound **7**.

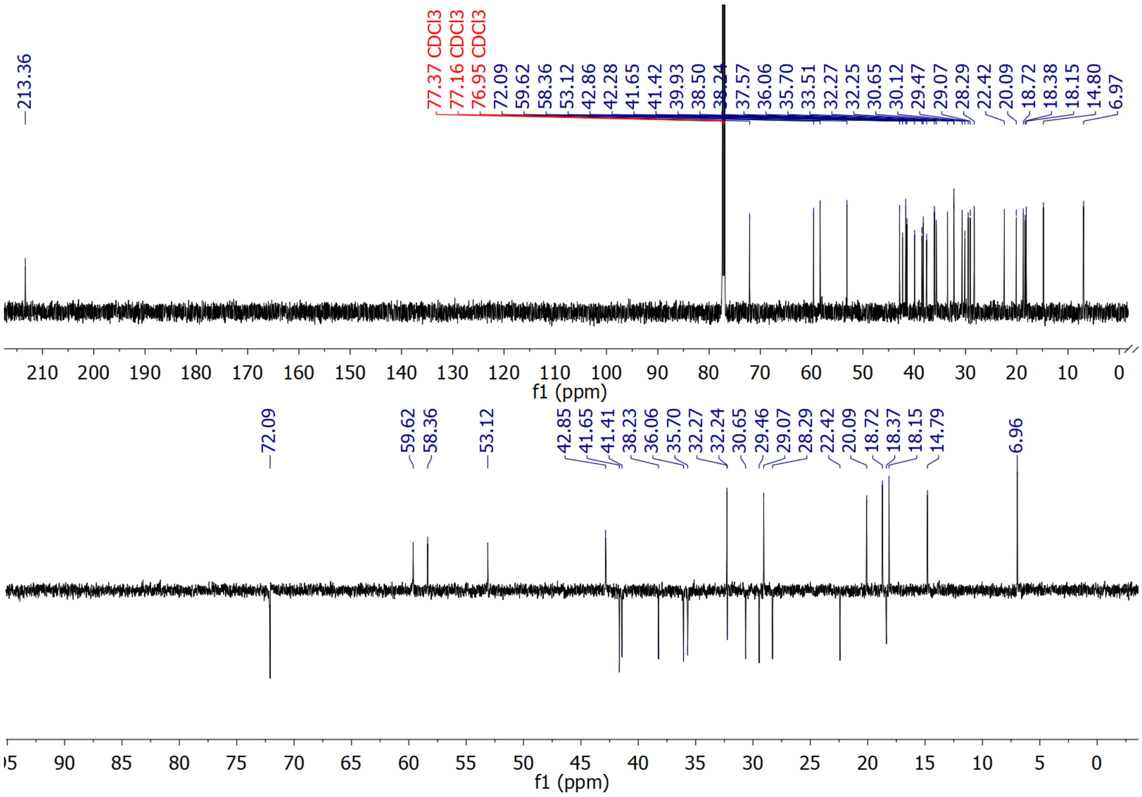

Figure S28. ^{13}C NMR and DEPT-135 spectra (150 MHz, $CDCl_3$) of compound **7**.

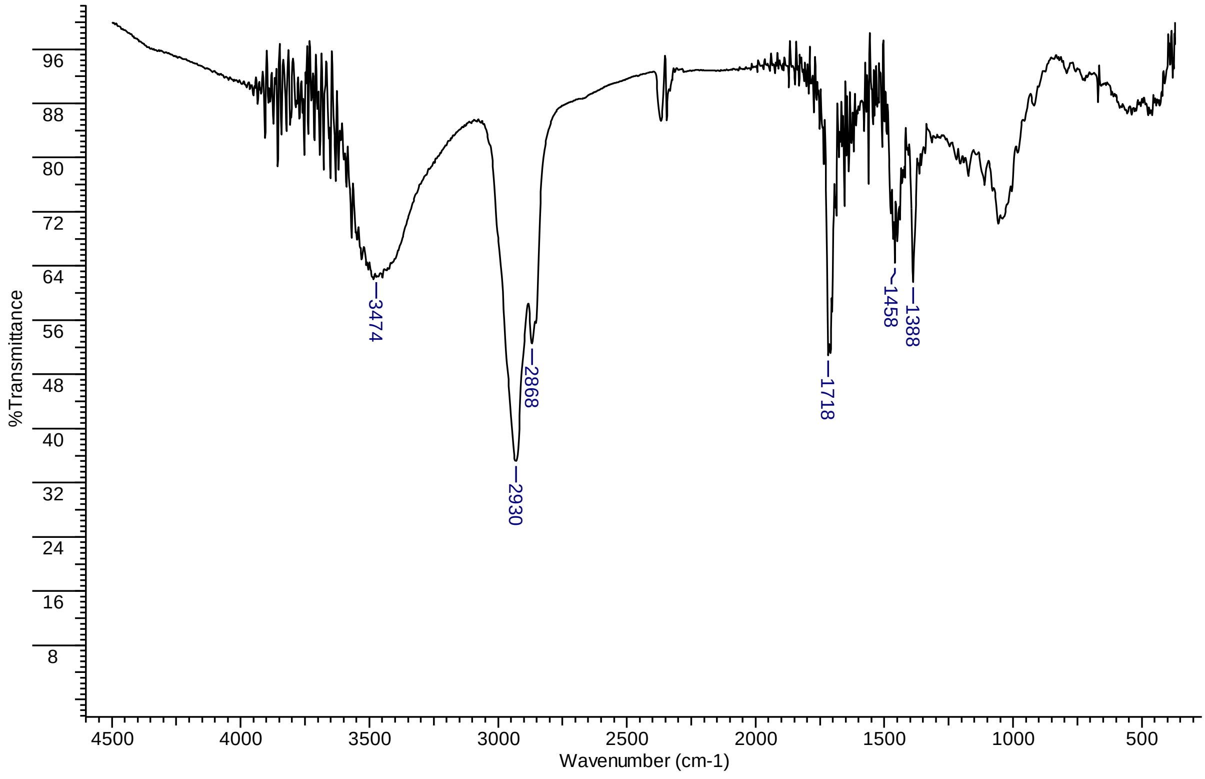

Figure S29. FTIR (KBr) spectrum of compound **8**.

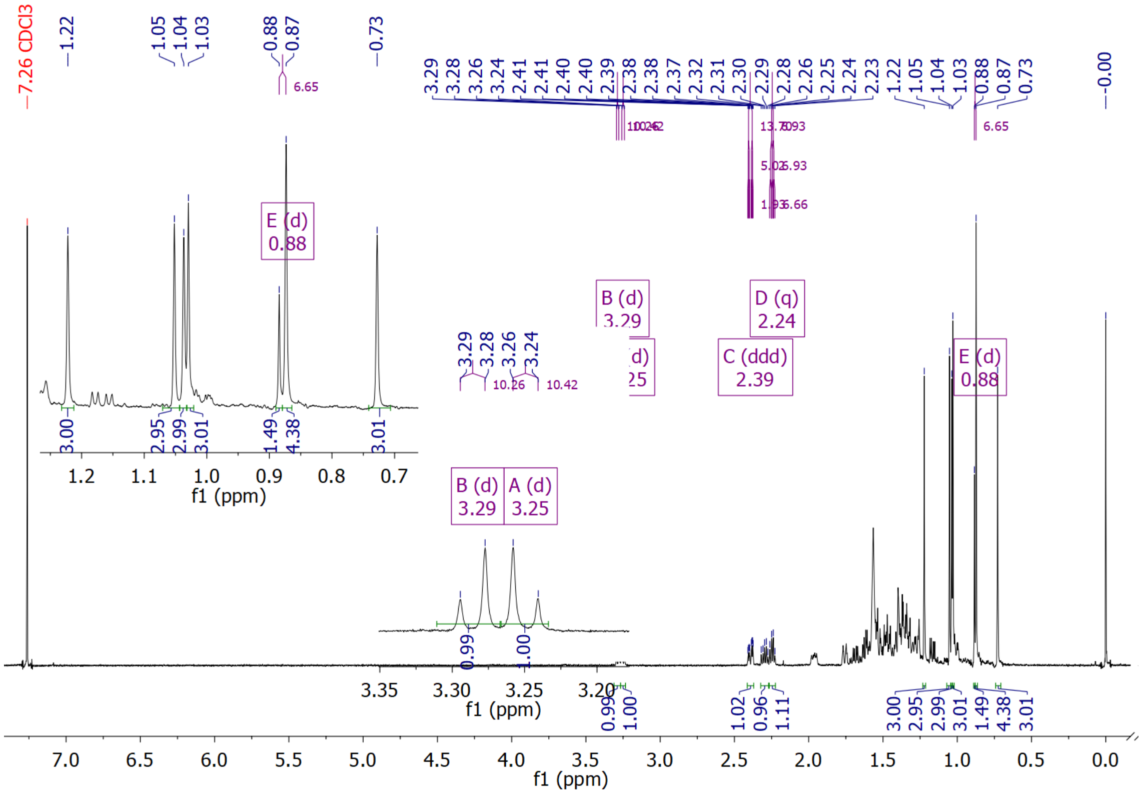

Figure S30. ^{1}H NMR spectrum (600 MHz, $CDCl_3$) of compound **8**.

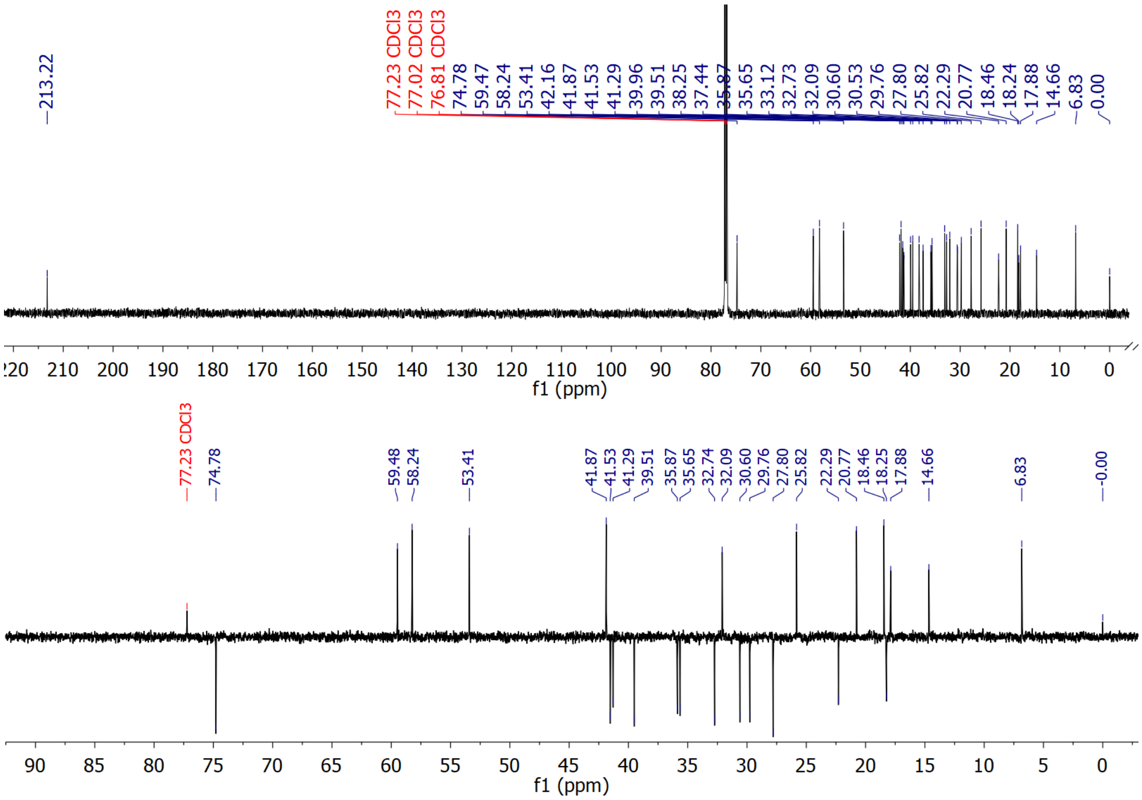

Figure S31. ^{13}C NMR and DEPT-135 spectra (150 MHz, $CDCl_3$) of compound **8**.

References

1. Klass, J.; Tinto, W. F.; *J. Nat. Prod.* **1992**, *55*, 16262.

2. Camargo, K. C.; de Aguilar, M. G.; Moraes, A. R. A.; de Castro, R. G.; Szczerbowski, D.; Miguel, E. L. M.; Oliveira, L. R.; de Sousa, G. F.; Vidal, D. M.; Duarte, L. P.; *Molecules* **2022**, *27*, 959.
